# Small molecules in the treatment of COVID-19

**DOI:** 10.1038/s41392-022-01249-8

**Published:** 2022-12-05

**Authors:** Sibei Lei, Xiaohua Chen, Jieping Wu, Xingmei Duan, Ke Men

**Affiliations:** 1 State Key Laboratory of Biotherapy and Cancer Center, West China Hospital, Sichuan University, Chengdu, 610041 People’s Republic of China; 2 Department of Pharmacy, Personalized Drug Therapy Key Laboratory of Sichuan Province Sichuan Academy of Medical Sciences & Sichuan Provincial People’s Hospital, School of Medicine, University of Electronic Science and Technology of China, Chengdu, 610072 China

**Keywords:** Drug development, Molecular medicine

## Abstract

The outbreak of COVID-19 has become a global crisis, and brought severe disruptions to societies and economies. Until now, effective therapeutics against COVID-19 are in high demand. Along with our improved understanding of the structure, function, and pathogenic process of SARS-CoV-2, many small molecules with potential anti-COVID-19 effects have been developed. So far, several antiviral strategies were explored. Besides directly inhibition of viral proteins such as RdRp and M^pro^, interference of host enzymes including ACE2 and proteases, and blocking relevant immunoregulatory pathways represented by JAK/STAT, BTK, NF-κB, and NLRP3 pathways, are regarded feasible in drug development. The development of small molecules to treat COVID-19 has been achieved by several strategies, including computer-aided lead compound design and screening, natural product discovery, drug repurposing, and combination therapy. Several small molecules representative by remdesivir and paxlovid have been proved or authorized emergency use in many countries. And many candidates have entered clinical-trial stage. Nevertheless, due to the epidemiological features and variability issues of SARS-CoV-2, it is necessary to continue exploring novel strategies against COVID-19. This review discusses the current findings in the development of small molecules for COVID-19 treatment. Moreover, their detailed mechanism of action, chemical structures, and preclinical and clinical efficacies are discussed.

## Introduction

**Fig. 1:**
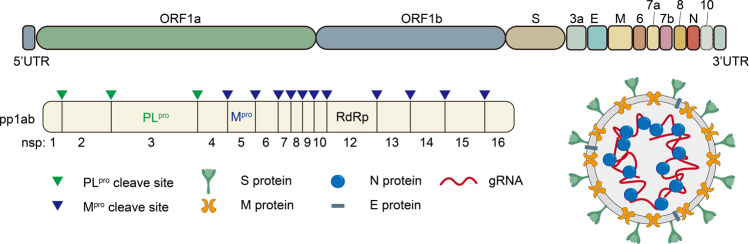
Schematic illustration of the genome of SARS-CoV-2 and its structure. The size of SARS-CoV-2 genome is close to 30 kb; it contains 14 open reading frames (ORFs) and encodes 29 proteins. Two ORFs, comprising approximately two-thirds of the genome, encode two polyproteins, which are digested by M protease (M^pro^) and Papain-like protease (PL^pro^) into 16 nonstructural proteins (nsps). Four ORFs encode a series of structural proteins, including the spike (S), membrane (M), envelope (E), and nucleocapsid (N) proteins

COVID-19, caused by severe acute respiratory syndrome coronavirus 2 (SARS-CoV-2), has led to more than 6 million deaths worldwide.^1^ SARS-CoV-2 is a betacoronavirus and possesses a positive-sense single-stranded RNA genome that contains 14 open reading frames (ORFs) (Fig. 1). Two ORFs encode polyproteins PP1a and PP1b.^2^ Four ORFs encode a series of structural proteins, including the spike (S), membrane (M), envelope (E), and nucleocapsid (N) proteins. In the SARS-CoV-2 lifecycle, S protein, which recognizes the human ACE2 receptor and is cleaved by host proteases, is responsible for virus binding and entry into host cells.^3,4^ Subsequently, M^pro^ and PL^pro^ are necessary for the production and function of non-structural proteins (NSPs). The key NSP RNA-dependent RNA polymerase (RdRp, also known as NSP12) catalyzes the synthesis of viral RNA and plays a central role in the lifecycle of SARS-CoV-2.^5–7^ Therefore, targeting these functional proteins is a rational strategy to inhibit infection and the replication of SARS-CoV-2. Infection with SARS-CoV-2 activates the host immune system, which may elicit a dysfunctional inflammatory response and cause organ damage.^8–10^ Therefore, therapeutic interventions targeting the immune system are also potential approaches for COVID-19 therapy.

Small molecules targeting specific signals and functions are widely applied in the treatment of diseases. Compared with biologics such as monoclonal antibodies and plasma products, small molecules are more flexible in binding with target molecules when acting as antagonist or agonist.^11,12^ Their lower production cost and higher stability also make them ideal therapeutic agents for both clinical and research applications. In parallel with the growing understanding of the pathogenic mechanisms of SARS-CoV-2 infection, small molecules from natural sources or those produced via chemical synthesis have demonstrated their immense therapeutic potential by intervening with various processes.^13–15^ The development of small molecules to treat COVID-19 has been achieved by several strategies, including computer-aided lead compound design and screening, natural product discovery, drug repurposing, and combination therapy. In this review, we present a comprehensive overview of the latest progress in the development of small molecule therapeutics for COVID-19 treatment. These therapeutic compounds are classified according to their chemical structures. The anti-COVID-19 molecular mechanisms are also discussed.

## COVID-19 therapeutic targets for small molecules

### RNA-dependent RNA polymerase (RdRp)

**Fig. 2:**
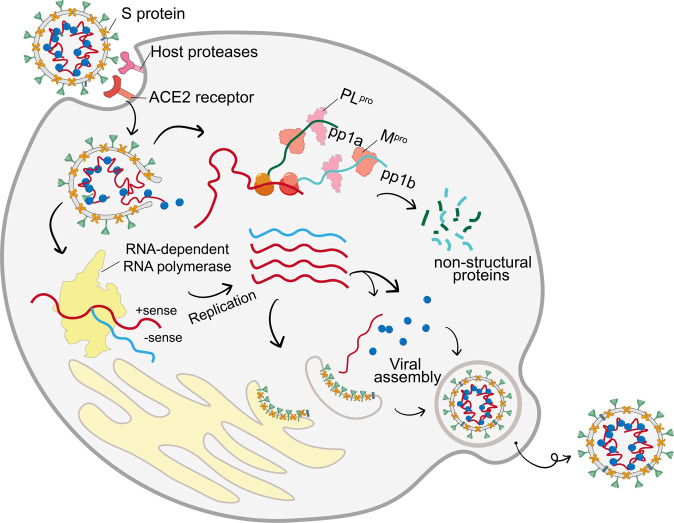
Lifecycle of SARS-CoV-2. The SARS-CoV-2 S protein recognizes the ACE2 receptor while being cleaved by the host proteases and entering into the target cells. Then, the gRNA is released and translated into pp1a and pplb, thereby being digested into the NSPs necessary for viral replication. Under the catalyzation of RdRp, new gRNAs are produced and encode the structural proteins to assemble the progeny virus

RdRp of SARS-CoV-2 is composed of NSP12 as the catalytic subunit and the NSP7–NSP8 complex as accessory subunits.^16–18^ RdRp is central to RNA transcription and viral replication, and may thus be an ideal target for anti-SARS-CoV-2 drugs (Fig. 2). The structural conformation of the SARS-CoV-2 RdRp complex is highly similar to that of SARS-CoV RdRp.^17,19^ NSP12 is classified into three domains: an N-terminal nidovirus RdRp-associated nucleotidyltransferase domain (residues 1–250), an interface region (residues 251–398), and the core RdRp domain (residues 399–932). NSP12 is formed by polymerase motifs A to G. These motifs are conserved in most RNA viruses.^17^ Studies of this RdRp domain have provided information on the role of these conserved motifs during RNA synthesis. Briefly, initial nucleotide recognition is mediated by positively charged Lys and Arg residues, which are located in motifs D and F of NSP12. The nucleotide flips into the active site through interaction with motifs A, B, and F to form a base pair with the template nucleotide, close to the active site. The incoming NTP forms a phosphodiester bond with the product RNA and after catalysis releases pyrophosphate. Then, the conformation of the active site immediately changes to an open state through a subtle rotation of motif A for the next nucleotide addition cycle.^20–22^ RdRp is the primary target of many existing antiviral nucleotide drugs. Based on its high conservation in diverse RNA viruses, repurposing of existing nucleotide drugs is an effective strategy that could shorten drug development time.^18,19^

**Fig. 3:**
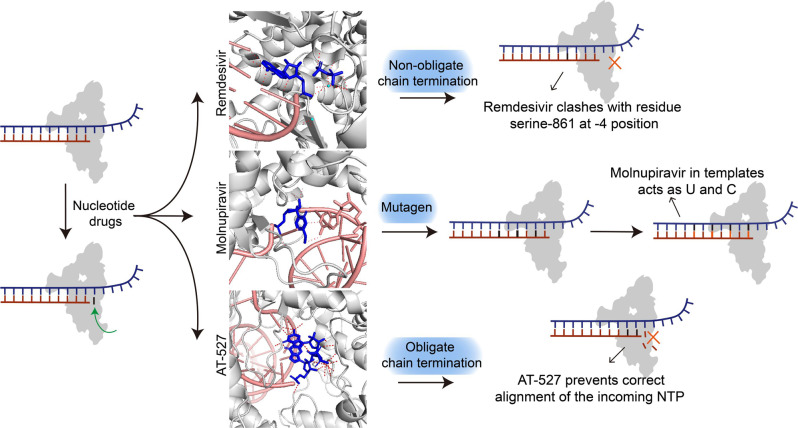
Antiviral mechanisms of nucleotide drugs. The triphosphate form of remdesivir acts as a non-obligate chain terminator to exert an inhibition effect (Protein Data Bank entries 7VB2^19^). The active form of molnupiravir can be directly incorporated into RNA as a substrate instead of cytidine triphosphate (C) or uridine triphosphate (U), thereby leading to mutated RNA products (Protein Data Bank entry 7OZU^38^). The triphosphate form of AT-527 (AT-9010) incorporates at the 3′ end of the RNA product, causing termination of RNA synthesis (Protein Data Bank entry 7ED5^51^)

The possible antiviral mechanism of nucleotide drugs is threefold; they can act as mutagens, as obligate chain terminators, and as non-obligate chain terminators (Fig. 3).^23,24^ Mutagens incorporated into RNA strands can cause permanent mutations.^25,26^ Obligate terminators lacking a 3-OH group will terminate RNA extensions immediately, while non-obligate chain termination usually proceeds when a drug contains both a natural base and a 3-OH on the sugar but has a modified ribose skeleton that disrupts translocation.^27,28^

Remdesivir was first developed for the prevention of the Ebola virus infection.^29–31^ It is a non-obligate chain terminator of SARS-CoV-2.^32^ A study conducted by Yin et al. revealed that the triphosphate form of remdesivir (GS-441524) mimics a nucleotide and is covalently linked to the replicating RNA, thus blocking further synthesis of SARS-CoV-2 RNA.^19^ Kokic et al. reported that incorporation of remdesivir into the RNA product could stop RNA synthesis after the addition of three more nucleotides.^33^ They showed that the stalling is caused by the C1ʹ-cyano group in the remdesivir ribose moiety. Insight into this non-obligate chain termination mechanism may facilitate the search for compounds with potential to interfere with SARS-CoV-2 replication.^16,34^

Molnupiravir, an orally available antiviral drug, is a mutagen of SARS-CoV-2.^35–37^ According to research reported by Kabinger et al., the active form of molnupiravir, beta-D-N4-hydroxycytidine triphosphate, can be directly incorporated into RNA as a substrate instead of cytidine triphosphate or uridine triphosphate, leading to mutated RNA products.^38^ Structural analysis of RdRp-mutated RNA indicated that beta-D-N4-hydroxycytidine triphosphate formed a stable base pair with G or A in the RdRp active region, thus escaping proofreading and synthesizing mutated RNA. Like molnupiravir, ribavirin abrogates viral RNA synthesis by incorporation into nascent RNA strands.^39–42^ Cheung et al. confirmed it is a mutagen for influenza virus by increasing the G-to-A and C-to-T mutation rates in vitro.^39^ The molecular docking study of Bylehn et al. indicated that it binds strongly at the active site of SARS-CoV-2 RdRp.^43^ However, their results revealed that ribavirin does not bind the nucleotide on the complementary strand as effectively and seems to act by a different mechanism.

Favipiravir is another inhibitor of RdRp with two possible mechanisms of action.^44–47^ Shannon et al. demonstrated its active form could result in SARS-CoV-2 lethal mutagenesis by incorporation into the nascent viral RNA by error-prone SARS-CoV-2 RdRp, provoking C-to-U and G-to-A mutations in the SARS-CoV-2 genome.^48^ This mutagen mechanism of favipiravir was also reported by Peng et al.^49^. A study conducted by Naydenova et al. indicated that favipiravir could suppress the replication of SARS-CoV-2 RNA in the presence of natural nucleotides by weak incorporation into the RNA prime strand.^50^ They revealed that favipiravir–RTP represents an unusual, non-productive binding mode at the catalytic site of SARS-CoV-2 RdRp, thus inducing non-obligate chain termination.

The obligate chain terminator AT-527 is a guanosine nucleotide analog that serves as an orally available prodrug with inhibitory effects on hepatitis C virus (HCV) RdRp.^51,52^ Shannon et al. reported a 2.98 Å cryo-EM structure of the SARS-CoV-2 RdRp–RNA complex, showing the triphosphate form of AT-527 (AT-9010) bound at three sites of NSP12.^51^ Their results showed that after AT-9010 is incorporated at the end of the RNA product strand, its modified ribose group will prevent correct alignment of incoming NTP, thereby causing obligate chain termination.

Due to the conserved structure of RdRp, the effects of several molecules interfering with other viral RdRps against RdRp of SARS-CoV-2 were also studied.^17,53^ For example, sofosbuvir is an oral nucleoside that is used to treat chronic HCV infection.^54–57^ Appleby et al. indicated that the metabolized form of sofosbuvir could be recognized by HCV RdRp (NS5B) and incorporated into the growing chain. The presence of fluoro and methyl modifications at the 2′ position promotes non-obligate chain termination of HCV RNA.^58^ Enzymatic assays demonstrated that sofosbuvir acts as a competitive inhibitor of SARS-CoV-2 RdRp,^59^ revealing it might act as a non-obligate terminator. Another molecule, galidesivir, was initially designed to inhibit filovirus RNA polymerase activity indirectly through non-obligate RNA chain termination.^60–62^ It exhibited activity against numerous viruses, including yellow fever virus, dengue virus, Japanese encephalitis virus, West Nile virus, zika virus, and tick-borne encephalitis virus, in cell cultures and animal models.^63^ Molecular docking assays also revealed galidesivir is attached to the catalytic center of SARS-CoV-2 RdRp, and its binding mechanism needs to be further studied.^61^

### Main protease (M^pro^)

SARS-CoV-2 M^pro^ (also named NSP5 or 3C-like protease) is a key enzyme that plays a vital role in viral replication and transcription.^64–66^ After membrane fusion, genomic RNA (gRNA) of SARS-CoV-2 is released into the cytosol of the target cell (Fig. 2). The gRNA of SARS-CoV-2 contains two large replicase ORFs, ORF1a and ORF1b. These ORFs encode two N-terminal polyproteins, PP1a and PP1ab, respectively.^67^ M^pro^ mainly digests both polyproteins at more than 11 conserved sites, thus helping to release NSPs.^68^ These NSPs are involved in the production of subgenomic RNA, encoding four major structural proteins and other helper proteins.^69–71^ Since no human protease has a structure similar to that of M^pro^, it is an attractive target for SARS-CoV-2 treatment.^72^ The SARS-CoV-2 M^pro^ crystal structure revealed it is a homodimer containing two protomers (promoters A and B), and each protomer is composed of three domains.^68,73,74^ The substrate binding site was located between (i) Domains I and II and (ii) Domain III. It regulates the dimerization of M^pro^, which is necessary for its catalytic activity.^72^ The active sites of M^pro^ between Domains I and II are composed of four sites (S1′, S1, S2, and S4), which often accommodate four fragments (P1′, P1, P2, and P3, respectively) of inhibitors.^68,73–75^ Among them, covalent linkage with the Cys-145 residue in the S1′ site is beneficial for the activity of inhibitors.^70,76^ Non-covalent SARS-CoV-2 M^pro^ inhibitors binding with M^pro^ in different patterns have also became clinical candidates for treating SARS-CoV-2.^77,78^

**Fig. 4:**
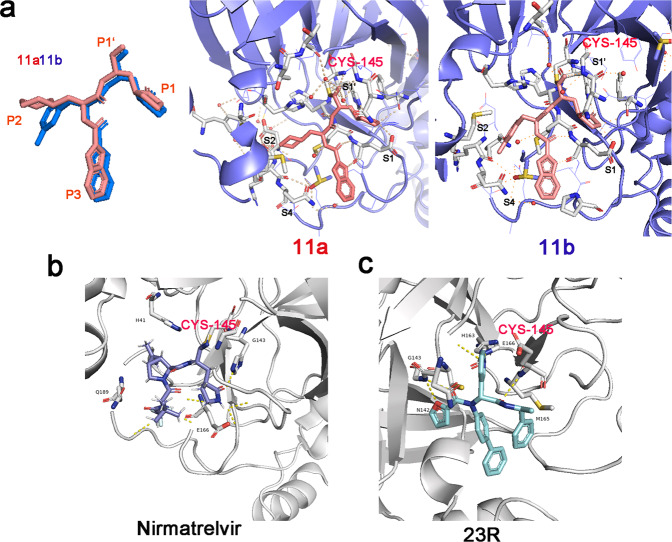
Different binding models of inhibitors in complex with SARS-CoV-2 M^pro^. **a** Binding models of inhibitors 11a and 11b complexing with SARS-CoV-2 M^pro^ (the Protein Data Bank entries for SARS-CoV-2 M^pro^ complexing with 11a and 11b are 6LZE and 6M0K, respectively^70^) **b** Binding model of inhibitor nirmatrelvir in complex with SARS-CoV-2 M^pro^ (Protein Data Bank entry 7RFW).^84^ **c** Binding model of non-covalent inhibitor 23R in complex with SARS-CoV-2 M^pro^ (Protein Data Bank entry 7KX5^77^)

M^pro^ always accommodates four fragments—P1′, P1, P2, and P3—which occupy the S1′, S1, S2, and S4 pockets of M^pro^, respectively. Following this rule, novel molecules against SARS-CoV-2 M^pro^ were developed by structure-based design methods. For example, Dai et al. designed and synthesized two lead compounds (11a and 11b) targeting M^pro^^70^ (Fig. 4). In their design, an aldehyde was selected as a new warhead along with an (S)-γ-lactam ring in order to form a covalent bond with cysteine. A cyclohexyl or 3-fluorophenyl was introduced in P2, while an indole group was introduced into P3. The resulting 11a and 11b were covalently bound to Cys-145 of M^pro^ according to the X-ray crystal structures of their complexes with SARS-CoV-2 M^pro^. Qiao et al. designed new inhibitors by fixing P1 as an optimal fragment, using P2 that was derived from either boceprevir or telaprevir and allowing P3 to change.^79^ According to their results, one of the most potent compounds, MI-23, covalently bound to the catalytic residue Cys-145 of SARS-CoV-2 M^pro^ as expected. The binding pattern of the representative compound MI-23 with M^pro^ is consistent with its design concept. Based on the structure of ML188(R), a non-covalent inhibitor of SARS-CoV M^pro^, Kitamura et al. proposed a strategy for designing the SARS-CoV-2 M^pro^ inhibitor and obtained a novel M^pro^ inhibitor 23R with high specificity to SARS-CoV-2 and SARS-CoV M^pro^.^77^ Furthermore, they designed covalent SARS-CoV-2 M^pro^ inhibitors Jun9-62-2R and Jun9-57-3R using novel cysteine reactive warheads to improve the target specificity of aldehyde warhead.^80^ To optimize oral bioavailability of M^pro^ inhibitors, Quan et al. chose alpha-ketoamide as warhead P1’, and P1, P2, and P3 were fixed as pyridine, tert-butylbenzene, and tert-butyl, respectively, similar to the groups in ML188.^81^ The resulting compound Y180 showed high oral bioavailability in mice and efficiently protected transgene mice from SARS-CoV-2 and variant infection.

Besides the rational design of novel compounds, several SARS-CoV-2 M^pro^ inhibitors were discovered by optimizing existing M^pro^ inhibitors through drug design.^82,83^ The drug PF-07321332, more commonly known as nirmatrelvir, was optimized from the SARS-CoV M^pro^ inhibitor PF-00835231.^84^ Meanwhile, Zhang et al. optimized the structure of the alpha-ketoamide M^pro^ inhibitor 11r to increase its half-life and solubility and reduce its interaction with plasma proteins.^72^ Then, the authors replaced the P2 cyclohexyl moiety with a small cyclopropyl to increase the antiviral activity by scarifying the broad-spectrum nature.^65^ The molecule 13b was located in the substrate binding cleft of M^pro^ and interacted with the Glu-166 residue, thus disturbing the correct shape of the S1 pocket and inactivating the enzyme.^72,85^ Kenller et al. presented the design and characterization of three hybrid reversible covalent SARS-CoV-2 M^pro^ inhibitors named BBH-1, BBH-2, and NBH-2 by splicing the SARS-CoV protease inhibitors boceprevir and narlaprevir.^86^ By substituting the ketoamide group of boceprevir with the keto-benzothiazole moiety or introducing the nitrile warhead, they directed the warhead into the oxyanion hole. Then, they substituted the P1 group of boceprevir and narlaprevir with a Gln-mimic γ-lactam, thereby synthesizing the hybrid reversible covalent inhibitors BBH-1, BBH-2, and NBH-2. A study by Amporndanai et al. indicated that ebselen and its derivative MR6-31-2 solely bind at the M^pro^ catalytic site by donating a selenium atom, forming a covalent bond and blocking the His-41 and Cys-145 catalytic dyad.^87^

The three-dimensional structure of SARS-CoV-2 M^pro^ is highly similar to that of SARS-CoV M^pro^.^72,88–90^ Therefore, repurposing of drugs is a good strategy to develop drugs against SARS-CoV-2. Two SARS-CoV M^pro^ inhibitors, GRL-1720 and 5 h, have shown anti-SARS-CoV-2 activity.^91–93^ According to X-ray structural analysis, 5 h fully occupies all binding pockets and is stabilized by six direct hydrogen bonds with the residues inside the binding groove of SARS-CoV-2 M^pro^, and covalent bonds are formed between 5 h and the Cys-145 residue.^91^ Su et al. reported that myricetin inhibits SARS-CoV-2 M^pro^.^94^ According to a crystal structure of the SARS-CoV-2 M^pro^–myricetin complex, an exact covalent bond can be observed between the sulfur atom of Cys-145 and the C6’ atom of the pyrogallol group of myricetin, revealing the potential of pyrogallol as an alternative warhead of an M^pro^ inhibitor. High-throughput screens were also applied to repurpose molecules with potential inhibitory effects on SARS-CoV-2 M^pro^.^95–97^ For example, Günther et al. applied X-ray fragment screening experiments with approved drugs and drugs in clinical trials, and identified 37 compounds that bind to M^pro^ .^88^ Moreover, they obtained structural evidence for interaction of seven compounds at active and allosteric sites of M^pro^, and identified two allosteric sites representing attractive targets for drug development. Another high-throughput screening study was conducted by Drayman et al. on a library of 1900 clinically safe drugs against OC43, which is also a betacoronavirus.^98^ As a result, they identified the most potent SARS-CoV-2 M^pro^ inhibitor, masitinib, and characterized the mechanism by X-ray crystallography. Virtual high-throughput screening methodology was also applied in identifying novel inhibitors from a large collection. Jin et al. assayed more than 10000 compounds through structure-based virtual screening and high-throughput screening, and identified ebselen as a promising inhibitor of SARS-CoV-2 M^pro^.^68^

### Papain-like protease (PL^pro^)

**Fig. 5:**
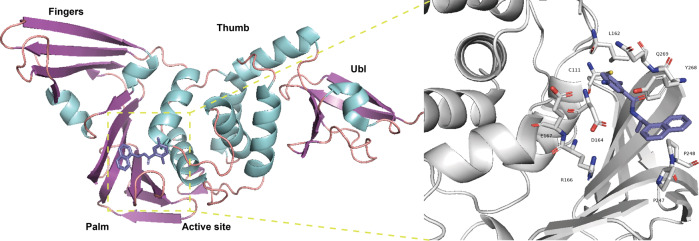
Cartoon structure of SARS-CoV-2 PL^pro^ in complex with GRL0617 (Protein Data Bank entry 7CJM)^112^ and the key residues in the PL^pro^ domain (Protein Data Bank entry 7JRN)^111^

PL^pro^ (NSP3) is an important coronavirus enzyme that digest polyproteins by recognizing the conserved sequence LXGG, thus generating a functional replicase complex which enables viral spread^99–101^ (Fig. 2). In addition, it is implicated in both the ubiquitination and inhibition of ISGylation on host proteins as an evasion mechanism against host antiviral immune responses.^102–104^ Shin et al. demonstrated that SARS-CoV-2 PL^pro^ prefers to cleave the conserved LRGG motif at the C-terminus of interferon-stimulated gene 15 (ISG15), which attenuates type I interferon immune responses elicited by viral infection.^105^ This dual functionality of PL^pro^ makes it an attractive antiviral target for SARS-CoV-2 treatment. PL^pro^ has four subdomains: the ubiquitin-like domain, the Thumb domain, the Finger domain, and the Palm domain (Fig. 5).^105^ The substrate binding pockets are located at the interface of the Palm and Thumb domains, which include a conserved catalytic triad of Cys-111. The other two core residues, Phe-69 and Val-66, mediate interactions of PL^pro^ with ISG15.^105^ Substrates accessing the active site are regulated by a flexible blocking loop 2 (BL2).^99^ The key Tyr-268 residue on BL2 is vital for regulating the function of the enzyme.^102^ In addition, the zinc finger domain comprises four cysteines which also contribute to the structural integrity and protease activity of PL^pro^.^106–108^ These sites are hotspots on PL^pro^, which have led to the discovery of drug leads with clinical potential for COVID-19 treatment.

GRL-0617 is a non-covalent inhibitor of SARS-CoV PL^pro^, and it exhibited inhibitory effects against SARS-CoV-2 in vitro.^103,109,110^ Gao et al. demonstrated that GRL0617 not only occupies the substrate pockets, but also induces closure of the BL2 loop and narrows the substrate binding cleft, thus preventing binding of the LXGG motif of the substrate.^99^ This BL2 conformational change was also observed by Ma et al. through X-ray co-crystal analysis of PL^pro^ complexed with GRL0617 (Fig. 5).^111^ Further, Shin et al. reported that GRL-0617 treatment of SARS-CoV-2-infected cells led to a marked increase in IRF3 ISGylation and significantly rescued the expression of IFN-responsive genes.^105^ According to Fu et al., GRL0617 blocks the binding of the ISG15 LRGG C-terminus to PL^pro^, thus interfering with cleavage of ISG15.^112^ Moreover, through a high-throughput screening and subsequent lead optimization, they identified two PL^pro^ inhibitors, Jun9-72-2 and Jun9-75-4. Both inhibitors demonstrated improved enzymatic inhibition and antiviral activity compared to GRL0617. In addition, Zhao et al. identified SARS-CoV-2 PL^pro^ inhibitors by high-throughput screening.^108^ They found that YM155, an anticancer drug candidate, efficiently inhibited the activity of SARS-CoV-2 PL^pro^. By analyzing crystal structures of SARS-CoV-2 PL^pro^ and its complex with YM155, they found that YM155 simultaneously targets the substrate binding pocket, the ISG15 binding site, and the zinc finger motif of enzyme.

Based on substrate specificity and the structure of SARS-CoV-2 PL^pro^, rational design of compounds would greatly facilitate the development of novel PL^pro^ inhibitors.^100^ For instance, by using a Hybrid Combinatorial Substrate Library, Rut et al. revealed the molecular rules governing PL^pro^ substrate specificity, and designed and biochemically characterized potent inhibitors (VIR250 and VIR251) with high selectivity for SARS-CoV-2 PL^pro^.^100^ Further, they found that both inhibitors could selectively inhibit the activities of PL^pro^ in both SARS-CoV and SARS-CoV-2. This revealed a high level of sequence and structural similarity between these PL^pro^ in the substrate binding pocket. The crystal structures of VIR250 and VIR251 in complex with SARS-CoV-2 PL^pro^ reveal they inhibit the enzyme by forming a covalent link with the Cys-111 residue and provide a structural basis for the observed substrate specificity profiles. Osipiuk et al. synthesized six naphthalene-based compounds derived from GRL0617. Five of them are further amine-functionalized derivatives of GRL0617, and one is a simplified variant of GRL0617 without a chirality center.^112,113^ All these compounds exhibited inhibition activities of PL^pro^, and the crystal structure indicated these inhibitors bind to protease S4/S3 sites, thus blocking peptide recognition. Shan et al. also synthesized a series of reported ScoV PL^pro^ inhibitors (11–13) that partially resemble GRL0617 with a shared naphthyl subunit.^114^ Co-crystal structure analysis of SARS-CoV-2 PL^pro^-12 revealed 12 occupies a pocket between the S1 position and the catalytic position of SARS-CoV-2 PL^pro^, and the three hydrophobic rings of 12 are engaged simultaneously with the phenyl ring of Tyr-268, thus closing the binding pocket.^114^

### Spike glycoprotein (S protein) and angiotensin-converting enzyme 2 (ACE2)

**Fig. 6:**
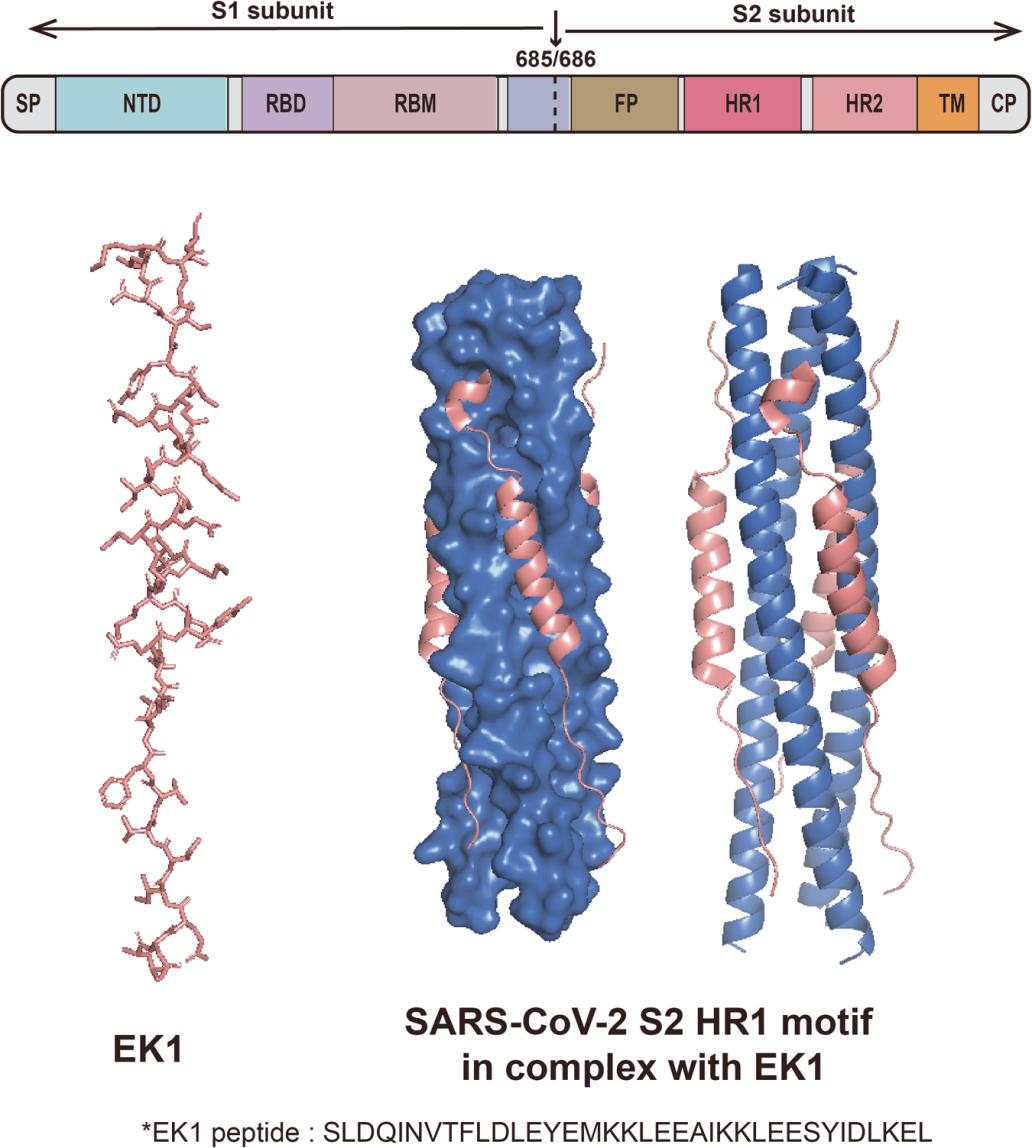
S2 subunit of SARS-CoV-2 S protein involves the HR1 and HR2 trimers to form a 6-HB domain. The binding model of the EK1 inhibitor in complex with the HR1 motif is presented (Protein Data Bank entry 7C53)^679^

SARS-CoV-2 virus entry into host cells depends on the viral S protein.^115–117^ In brief, the S protein recognizes the peptidase domain (PD) of the ACE2 receptor in host cells (Fig. 2). This initiates recognition of the virus and host cell receptor–viral membrane fusion.^118–120^ It was thought that targeting the virus entry process is more advantageous than targeting the subsequent stages of the SARS-CoV-2 lifecycle, thus many efforts have been made to find inhibitors blocking this process.^121–123^ Small molecules targeting the S protein, ACE2, and the S protein–ACE2 complex were found to potentially inhibit SARS-CoV-2 infection.^124,125^ The SARS-CoV-2 S protein consists of two subunits; S1 comprises the receptor binding domain (RBD) and S2 is responsible for viral membrane fusion.^126–130^ Previous studies revealed that the high affinity between the S protein RBD and the human ACE2 receptor could partially explain the efficient transmission of SARS-CoV-2 among humans.^131–133^ The structure of the SARS-CoV-2 RBD was found to have more ACE2-interacting residues than the SARS-CoV RBD.^119^ Compensating mutations in the S protein RBD of further variants (especially the Delta and Omicron variants) possibly account for their heightened transmissibility and immune evasion.^134,135^ Thus, interference with binding between them is beneficial for viral inhibition. A six-helical bundle (6-HB) structure of S2 conjuncts the viral and cell membranes for a fusion reaction.^136^ Blocking the 6-HB domain is considered effective for developing fusion inhibitors EK1 (Fig. 6).^137,138^ In human ACE2, Lys-31 and Lys-353 are sensitive to the RBD.^139^ Its glycosylation sites Asn-90 and Asn-322 also demonstrated the ability to interfere with S protein binding in a recent study.^140^ Glycosylation of asparagine residues within the RBD is an important mediator of ACE2 binding.^141^

The effects of molecules binding with S protein against SARS-CoV-2 were investigated. A previous study revealed that the RBD of the S protein of SARS-CoV-2 recognizes oligosaccharides containing sialic acid.^142^ Based on this, Petitjean et al. investigated the biophysical properties of S1 subunit binding to sialic acids or 9-O-acetylated sialic acid (9-AcSA) using force–distance (FD) curve-based atomic force microscopy.^143^ Then, they designed novel blocking molecules with various topologies and carrying multiple salic acid or 9-AcSA residues. They reported that 9-AcSA-derived porphyrin has strong inhibitory effects on SARS-CoV-2. Yi et al. searched for S protein RBD inhibitors by screening compounds from the Chinese herbal medicine licorice.^144^ They found that glycyrrhetinic acid (GA) and licorice saponin A3 target the S protein RBD, and Tyr-453 is a key residue for the affinity of triterpenoids with the S protein RBD. Another strategy to inhibit SARS-CoV-2 S protein is to disrupt the disulfide pairs of RBD.^145,146^ Disulfide bond formation is central to the dynamic structure of many viral receptor binding and entry/fusion proteins.^147^ The SARS-CoV-2 S protein RBD contains four disulfide pairs, which may interact with thiol-based reducing agents.^146,148^ Shi et al. reported that the preclinical thiol-based reducing agents P2110 and P2165 target a conserved hydrophobic binding pocket in the RBD, thus inhibiting SARS-CoV-2 infection.^146^ In detail, proteomic and reactive cysteine mapping showed that the disulfide pairs Cys-379–Cys-432 and Cys-391–Cys-525 are redox-sensitive and can be reduced by P2110 and P2165. A significant conformational change of the RBD was observed after reduction of both disulfide pairs. They also indicated that P2110 and P2165 could modulate the extracellular redox poise required for SARS-CoV-2 entry into cells, which is beneficial for preventing viral infection.

Besides finding molecules with inhibitory effects on the S protein, studies focused on finding molecules which can inhibit the RBD–ACE2 interaction.^149–151^ For example, Pei et al. applied a computer-aided approach based on the RBD binding residues on ACE2 to design ultrashort peptide inhibitors against SARS-CoV-2.^152^ Based on the critical residues of ACE2, they initially obtained the peptide inhibitor SI1. Then, using a “docking–activity test–molecular simulation–sequence improvement” scheme, they successfully obtained ultrashort peptides SI5α and SI5α-b, which had significantly higher activity. By analyzing the binding sites of ultrashort peptides to RBD, the residues from Glu-484 to Tyr-505 on the RBD were determined as the “binding pocket” in this study, which may be helpful for the design of RBD inhibitors or antibodies. A similar computer-aided strategy for the identification of novel inhibitors disrupting the RBD–ACE2 interaction was reported by Gupta et al. In their study, machine learning classifiers were applied for the prediction of new small molecular modulators of the SARS-CoV-2 S protein RBD–ACE2 interaction. Using this RBD: hACE2 predictor, they identified more than 300 novel small molecule scaffolds that can be repurposed for SARS-CoV-2. Panda et al. took the structure-based drug design approach for screening inhibitors with an affinity against M^pro^ and S protein.^153^ Molecular docking simulations indicated that the obtained molecule, PC786, has a binding affinity toward the RBDs of all the chains in the trimeric S protein. Their protein–protein interaction analysis revealed that conformational changes occur when PC786 interacts with the RBD–ACE2 complex, revealing that the binding of PC786 with S protein substantially affects S protein binding to the ACE2 domain. Lee et al. showed that both Etravirine and Dolutegravir preferentially bind to primary ACE2-interacting residues on the RBD domain, implying that these two drugs may inhibit attachment of SARS-CoV-2.^154^ Xiong et al. showed that the novel inhibitors DC-RA016 and DC-RA052 have the ability to interfere with the SARS-CoV-2 S protein RBD–ACE2 interaction, thus playing an anti-SARS-CoV-2 role.^155^

### Host proteases

After binding to the ACE2 receptor of host cells, S protein needs to be activated by host protease at the putative cleavage site located at the boundary of the S1 and S2 subunits, thus exposing the S2 subunit for viral entry (Fig. 2).^128,156,157^ This cleavage is performed by host cells proteases, including serine protease transmembrane protease, serine 2 (TMPRSS2), cysteine protease cathepsin L (CTSL), and the arginine protease furin.^54,121,158^ TMPRSS2 was thought to play an essential role in SARS-CoV-2 viral entry.^159–161^ It enables rapid endosome-independent virus entry of SARS-CoV-2 into the cells (within 10 min).^162^ CTSL also enhances SARS-CoV-2 infection in both human cells and human ACE2 transgenic mice.^163–165^ CTSL is critical for SARS-CoV-2 entry via endocytosis during infection.^157^ The furin cleavage site also has a critical role in SARS-CoV-2 infection,^164,166–168^ since a study has revealed that its cleavage site at the S1/S2 boundary is essential for S-protein-mediated cell–cell fusion and entry into human lung cells.^168^ Based on these observations, inhibitors of TMPRSS2, CTSL, and furin were identified as promising therapeutical agents for COVID-19 treatment.^169^

The structure of TMPRSS2 is characterized by an N-terminal cytoplasmic domain, a transmembrane domain, a class A LDL receptor domain, a scavenger receptor cysteine-rich domain, and an activation domain linked to a serine protease domain via a disulfide bond.^54,159,170^ Since no crystal structure of TMPRSS2 is available, repurposing or optimizing inhibitors against well-known serine proteases may facilitate the discovery of effective TMPRSS2 inhibitors against SARS-CoV-2.^170–172^ For example, Sun et al. identified structurally similar serine proteases using a structure-based phylogenetic computational tool to find potential inhibitors of TMPRSS2.^173^ According to their computational results, six serine peptidases, including kallikrein-related B1, had a high structural similarity to the TMPRSS2 S1 protease domain. The kallikrein-related B1 inhibitor avoralstat with high potential to be repurposed for COVID-19 therapy was identified. In addition, based on a previously designed peptidomimetic tetrapeptide with inhibitory activity against matriptase, Shapira et al. developed a small library of peptidomimetic compounds to screen for inhibitors of TMPRSS2.^174^ Through the screening process, they found that N-0385, containing a ketobenzothiazole warhead, inhibits TMPRSS2. Then, by building a homology model of TMPRSS2 using the crystal structure of matriptase, they found that the catalytic Ser-441 residue of the enzyme forms a covalent bond with the warhead of N-0385. This contributes to its inhibitory activity against TMPRSS2. Rational structure-based drug design was also applied to discover TMPRSS2 inhibitors by Mahoney et al..^175^ Based on molecular docking studies using a published homology model of TMPRSS2 and substrate specificity data from PS-SCL, a set of ketobenzothiazole inhibitors of HGF-activating serine proteases (including HGF activator [HGFA], matriptase, and hepsin) were developed. After further optimization, they identified multiple potent inhibitors of TMPRSS2. Four of these analogs displayed activity at subnanomolar concentrations, both in the enzyme assay and in blocking the entry of VSV-SARS-CoV-2 chimeras into human Clau-3 epithelial lung cells. Besides blocking the cleavage function of TMPRSS2, molecules with the ability to reduce TMPRSS2 expression on host cells also drew attention for anti-COVID-19 research. A high-throughput screening using a library of 2560 FDA-approved or currently investigated clinical compounds was carried out by Chen et al. to identify small molecules that reduce TMPRSS2 expression.^176^ They found that halofuginone modulates TMPRSS2 levels through proteasomal-mediated degradation that involves the E3 ubiquitin ligase component DDB1- and CUL4-associated factor 1.

CTSL is a lysosomal cysteine protease. It contains an L domain of alpha-helices and an R domain of beta-sheets.^177–179^ Gallinamide A is a potent covalent inhibitor of several parasite-derived cysteine proteases, as well as human CTSL.^180,181^ Ashhurst et al. demonstrated that Gallinamide A and analogs could directly interact with CTSL and potently inhibit SARS-CoV-2 infection in vitro.^182^ Structure-based design of CTSL inhibitors was carried out by Phan et al. According to their report, good peptidyl substrates can be converted into CTSL inhibitors that are active at submicromolar concentrations by a single thioamide substitution in the peptide backbone.^169^ By designing and scanning several thioamide-stabilized peptide scaffolds, they found that the peptide RS1A inhibits CTSL activity with >25-fold higher specificity compared to the other cathepsins. According to computational modeling analysis, the P1 thioamide N–H group of the peptide interacts with the His-163 catalytic triad of CTSL. In a recent preprint reported by Frueh et al., an orally available CTSL inhibitor K777 exhibited anti-viral ability and efficiently reduced COVID-19-related pulmonary pathology in African green monkeys.^183^ Despite these achievements, the ubiquitous expression of CTSL raises concern about the side effects of CTSL inhibitors.^184^ Combined use of a CTSL inhibitor and other protease inhibitors or development of a CTSL inhibitor with multiple functions might be effective in preventing viral infection at a lower dose and in reducing side effects. Thus, Hu et al. found that calpain inhibitors II and XII, and GC-376 have a dual mechanism of action by inhibiting both viral M^pro^ and host CTSL in vitro.^185^ In addition, Sacco et al. found that M^pro^ inhibitors targeting the hydrophobic methionine side chain in the S1 pocket are also active against CTSL, which paved the way for the design of dual inhibitors that target both viral M^pro^ and host CTSL.^186^

Furin recognizes and cleaves a polybasic stretch of an RRAR motif in the S1/S2 boundary of S protein. It is worth noting that the cleavage site of furin was only identified in SARS-CoV-2, and not in other lineages of betacoronaviruses.^187–190^ Even Papa et al. indicated that knockout of furin significantly suppressed but not abolished SARS-CoV-2 S-protein-mediated cell–cell fusion.^191^ Johnson et al. revealed that RRAR cleavage site mutation attenuates SARS-CoV-2 pathogenesis in both hamster and K18-hACE2 transgenic mouse models.^167^ Peacock et al. found that SARS-CoV-2 virus lacking the S1/S2 furin cleavage site was shed to lower titers from infected ferrets and was not transmitted to cohoused sentinel animals, unlike the wild-type virus.^168^ Thus, Cheng et al. reported that two molecular inhibitors of furin, decanoyl-RVKR-chloromethylketone (CMK) and naphthofluorescein, significantly inhibited syncytium formation in S-protein-expressing cells and cytopathic effects (CPEs) in SARS-CoV-2-infected cells.^187^ According to their results, CMK abolished CPEs and decreased virus titer in the preinfection treatment experiments, while it did not decrease virus production and infectivity but only decreased CPEs in postinfection treatment. This revealed that CMK affects the viral entry stage of SARS-CoV-2, and that it likely ameliorates viral virulence and pathogenicity. In addition, another furin inhibitor, naphthofluorescein, showed affinity at the replication stage when the virus entered the cell downstream.^192,193^ Authors speculated CMK and naphthofluorescein might act differently for furin substrates located in different compartments. It remains to be clarified whether naphthofluorescein’s function depends on furin activity or other new targets. Paszti-Gere et al. revealed that another furin inhibitor, MI-1851, could exert anti-SARS-CoV-2 effects on cells by suppressing the cleavage of S protein.^194^

### Immune regulation

**Fig. 7:**
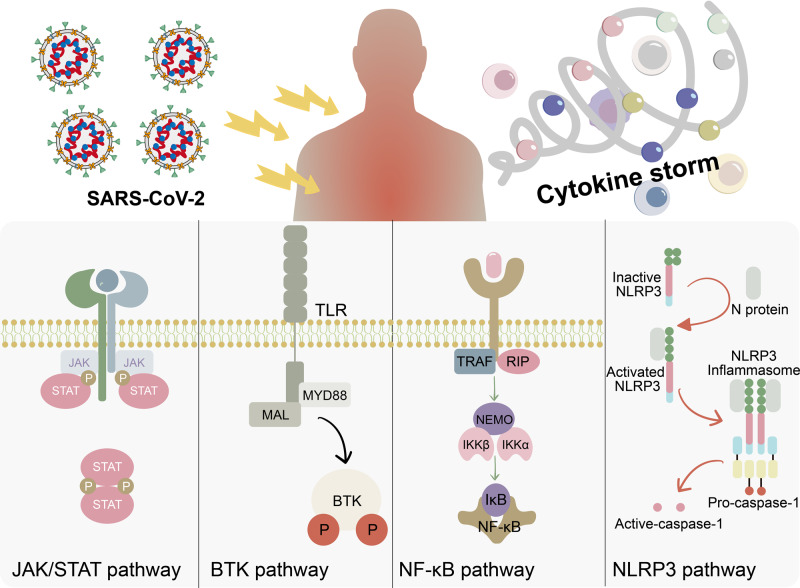
Illustration of SARS-CoV-2-induced immune responses and pro-inflammatory signaling pathways

SARS-CoV-2 infection activates both innate and adaptive immune responses, which may cause excessive inflammatory reactions and dysregulate the adaptive host immune response.^9,195–197^ Many studies have reported the influence of SARS-CoV-2 infection on the immune system of COVID-19 patients. In detail, lymphopenia was widely observed in patients with severe COVID-19.^67,198^ The proportion of lymphocytes is considered a reliable indicator of disease severity.^199^ In patients with severe COVID-19, the proportions of circulating CD4+ T cells, CD8+ T cells, B cells, and natural killer cells also decreased, while the proportions of immunosuppressive regulatory T cells were moderately increased in patients with mild COVID-19.^200–202^ Moreover, the levels of proinflammatory cytokines and chemokines (such as IL2, IL7, IL10, GSCF, IP10, MCP1, MIP1A, TNFα, and IL6) were significantly increased in severe patients.^198,201,203^ As a result of virus recognition, downstream immune-regulatory pathways such as nuclear factor κB (NF-κB), and Janus kinase (JAK)/Signal transducer and activator of transcription (STAT) pathways are activated (Fig. 7). These pathways are crucial for the antiviral response.^204–206^ In fact, mortality of COVID-19 patients is often caused by acute respiratory distress syndrome (ARDS), and ARDS is the result of dysregulated hyperinflammation in response to viral infection.^198,207,208^ Thus, various immune regulators were developed or repurposed for COVID-19 treatment (Fig. 3). Most immune regulators, such as glucocorticoids, function as inflammatory extinguishers. Here, we present the immunomodulatory mechanism of these molecules against COVID-19.

The JAK family consists of four non-receptor tyrosine protein kinases, JAK1, JAK2, JAK3, and TYK2.^209,210^ They are often activated when proinflammatory cytokines bind to their receptors, thus amplifying the inflammation caused by SARS-CoV-2 infection.^211^ So far, more than 50 cytokines that transmit their signals via JAK proteins have been identified.^212–214^ Based on this, it was recognized that JAK inhibitors could help to prevent the cytokine storm in severe COVID-19 patients.^213,215^ Baricitinib, a JAK1/JAK2 inhibitor, blocks the immune cascade and reduces SARS-CoV-2 replication in patients.^216–218^ According to a study conducted by Stebbing et al., type-1 interferons (IFNs), specifically IFN-α2, increased ACE2 expression in human liver cells could increase the viral load, and this induction is fully inhibited by the JAK inhibitor baricitinib.^219^ A study reported by Nystrom et al. indicated that baricitinib could block the cytokine-induced JAK/STAT/APOL1 signaling, which may rescue a severe kidney disease called COVID-19-associated nephropathy.^220^ Other JAK inhibitors, such as tofacitinib, ruxolitinib, and nezulcitinib, were also shown to exert effects against COVID-19 in clinical studies.^221–223^ According to a study of Yan et al., the JAK1/2 inhibitor ruxolitinib could normalize the SARS-CoV-2-induced complement hyperactivation in lung epithelial cells.^224^ Ruxolitinib was also clinically related to increased serum levels of inflammatory cytokines such as IL6 and the acute phase protein ferritin and cardiac improvement.^225^ Tofacitinib is a JAK1/JAK3 inhibitor known to be effective against cytokine signaling. It also inhibits JAK2 with a lower potency.^226–228^ Several studies indicated that it suppresses S-protein-potentiated STAT1 signaling and combats lung tissue-resident memory T cells which cause chronic inflammation and fibrosis when treating COVID-19.^210,229,230^

Bruton’s tyrosine kinase (BTK) is a cytoplasmic non-receptor tyrosine kinase (TK) expressed in all cells of the hematopoietic lineage, particularly B cells, mast cells, and macrophages.^231,232^ In addition, BTK-deficient macrophages are defective in expressing proinflammatory cytokines and preferentially polarize into anti-inflammatory M2 macrophages, even upon virus infection.^233^ A previous study indicated that inhibition of BTK attenuated neutrophil extracellular traps released into the lung with reduced levels of TNFα, IL1β, IL6, KC, and MCP-1 in mice after influenza A virus infection.^233^ Since cytokine release syndrome and resident macrophages may lead to pulmonary injury associated with COVID-19, Treon et al. reported that inhibitors of the BTK pathway may protect against pulmonary injury in COVID-19 patients.^234^ Chong et al. also suggested continuing BTK inhibitor treatment in patients who receive it for therapy of B cell malignancies with COVID-19, since the potential benefit of attenuation of M1 polarization to mitigate the immediate risk of COVID-19-related mortality outweighs the potential medium- to long-term risk of impaired humoral immunity.^235^ The BTK inhibitors ibrutinib, zanubrutinib, and acalabrutinib have been found to protect against pulmonary injury in a small group of participants infected with SARS-CoV-2.^232,236,237^

NF-κB is a proinflammatory transcription factor critically involved in both inflammatory and thrombotic responses.^238,239^ Its upregulation was widely observed in the development of SARS-CoV-2 infection.^240–243^ In addition, N protein and NSP5 of SARS-CoV-2 facilitate NF-κB hyperactivation, thus inducing inflammation.^244–246^ Therefore, NF-κB has become a potential immunotherapeutic target for COVID-19 treatment.^247–249^ Sharma et al. reported that curcumin could potently inhibit the inflammatory response elicited by SARS-CoV-2 S protein in cells by deactivating MAPK/NF-κB signaling.^250^ Lee et al. found that the NF-κB inhibitor pyrrolidine dithiocarbamate suppresses ACE2 protein expression in human lung cell lines, which indicates another potential mechanism by which NF-κB inhibitors may combat COVID-19.^251^

The Nod-like receptor family pyrin domain-containing 3 (NLRP3) inflammasome is activated when viral infection-associated pathogens are recognized by the innate immune system.^252,253^ Activation of the NLRP3 inflammasome pathway leads to release of the proinflammatory cytokines IL18 and IL1β, which mediate cytokine release and pyroptosis during lung injury and ARDS.^254–256^ Rodrigues et al. demonstrated that the NLRP3 inflammasome is activated in COVID-19 patients. Inflammasome-derived products such as IL18 in the serum were correlated with disease severity.^257^ A study of Pan et al. revealed that the N protein of SARS-CoV-2 promotes NLRP3 inflammasome activity and induces an excessive immune response.^258^ Therefore, inhibitors targeting the NLRP3 inflammasome might serve as drugs to treat COVID-19.^259^ A study conducted by Zeng et al. demonstrated that inhibition of the NLRP3 inflammasome by MCC950 alleviated excessive lung inflammation. Further, they showed that MCC950 could reduce COVID-19-like pathology in human ACE2 transgenic mice.^260^

## Structures of small molecule drugs for COVID-19 therapy

### Nucleoside/nucleotide analogs

**Fig. 8:**
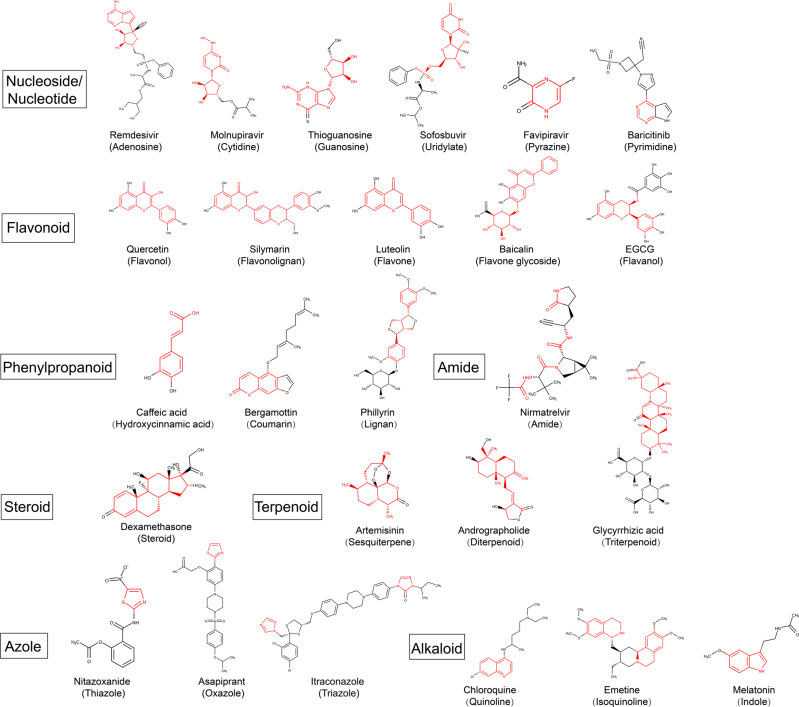
Chemical structure of representative small molecules and their backbone (labeled in red)

Nucleoside/nucleotide analogs were investigated widely in the area of antiviral drugs (Fig. 8).^261–263^ Generally, nucleoside/nucleotide analogs resemble naturally occurring nucleosides, and act as normal nucleotides, being recognized by viral polymerases or cellular enzymes, and prevent virus replication.^264,265^ Various nucleoside/nucleotide analogs have been applied for clinical antiviral therapies. Besides the first anti-HSV drug, acyclovir,^266^ other nucleoside/nucleotide analogs such as zidovudine against HIV, telbivudine against HBV, and sofosbuvir against HCV also exhibited specific therapeutic effects.^267–269^ Although achievements have been made in the area of DNA virus application, these analogs are still facing challenges in the treatment of infections with RNA viruses with higher spread and mutation rates. In the area of SARS-CoV-2, looking for nucleoside/nucleotide analogs is the preferred strategy, as no homolog of RdRp has been found in human cells. Since the RdRp of SARS-CoV-2 is conserved, exploring the anti-SARS-CoV-2 effects of pre-existing antiviral nucleoside/nucleotide analogs against the virus has been shown to be an effective way.^270,271^ Nucleobase analogs and double-stranded RNA (dsRNA) compounds with anti-SARS-CoV-2 effects will also be discussed in this section. Although they do not function by imitating nucleosides, those analogs and compounds interfere with viral infection by various mechanisms.

As a constituent of ATP and cAMP, adenosine participates in numerous processes in the human body.^272,273^ Therefore, numerous adenosine analogs have been synthesized against various diseases, including COVID-19. Among the existing adenosine analogs against COVID-19, the most investigated one is remdesivir. It was developed by Gilead to combat the Ebola virus, and it bears the structure of an adenine c-nucleoside modified by monophosphoramide and cyano groups.^274^ As a nucleotide prodrug, remdesivir is metabolized by the host cell to the pharmacologically active triphosphate to inhibit the activation of RdRp.^31^ In a study reported by Pruijssers et al., remdesivir exhibited a potent in vitro inhibition ability against SARS-CoV-2 replication in human lung cells and primary human airway epithelial cells.^275^ Its in vivo effect was also confirmed in SARS-CoV-2-infected rhesus macaques. Remdesivir treatment in rhesus macaques with COVID-19 efficiently prevented progression to pneumonia. Holshue et al. first reported its clinical application, which described an immediate improvement in clinical symptoms in the first confirmed case of SARS-CoV-2 after receiving remdesivir administration.^276,277^ However, according to a recent study conducted by Stevens et al., remdesivir resistance was observed in SARS-CoV-2 after 13 passages of co-culturing with GS-441525.^278^ Although it is encouraging that natural variants did not propagate remdesivir resistance mutations, this study emphasized that the extended use of remdesivir might increase the possibility for SARS-CoV-2 to adapt to remdesivir. It is worth noting that remdesivir is a prodrug of GS-441524, which has also been proved to be effective against COVID-19.^279,280^ GS-441524 is also developed by Gilead, which is the dephosphoramidated ribonucleoside parent nucleus of remdesivir.^281^ Pharmacokinetic analysis showed that GS-441524 is the predominant metabolite of remdesivir reaching the lungs. Based on its easy synthesis and high lung loads, Yan et al. claimed it is superior to remdesivir for COVID-19 treatment.^280^ Li et al. reported that GS-441524 effectively inhibited SARS-CoV-2 in three cell lines (Vero E6, Calu-3, and Caco-2).^282^ In addition, remdesivir can only be given intravenously, and there is a pressing medical need for oral antivirals. Xie et al. performed an in vitro and in vivo drug metabolism and pharmacokinetics assessment to examine the potential of GS-441524 as an oral drug.^283^ In further in vivo studies in CD-1 mice, GS-441524 displayed a favorable oral bioavailability of 57%. Due to these advantages, the first study of orally administered GS-441524 for COVID-19 in humans was started on January 1, 2021, and conducted by Copycat Sciences. The clinical results suggested the high safety and low toxicity of orally administered GS-441524 in healthy people.^284,285^ Although further clinical studies of the compound remain to be implemented, GS-441524 has potential as an oral drug for treatment of COVID-19. Further, another prodrug of GS-441524 named VV116 was developed by the Shanghai Institute of Materia Medica. VV116 is derived from GS-441524 by esterification of all three hydroxyl groups and replacing a hydrogen atom on the basic group with a D atom.^286^ Wu et al. reported that VV116 is highly effective in inhibiting SARS-CoV-2 replication in cell-based and animal models.^287^ A clinical study of VV116 showed that it has good safety and efficacy.^288^ Moreover, studies have shown that VV116 exhibits antiviral activity against the Alpha, Beta, Delta, and Omicron variants with high oral bioavailability and good chemical stability.^289^ Two international phase II/III clinical trials of VV116 are underway. Besides remdesivir and its analogs, another adenosine analog, galidesivir, also is notable as an anti-SARS-CoV-2 drug. Galidesivir was developed by BioCryst Pharmaceuticals and was originally intended as a drug for HCV treatment.^63^ Unlike the pyrrolotriazine group in the abovementioned compounds, galidesivir bears a pyrrolopyrimidine group as its nucleobase. A molecular docking study conducted by Aftab et al. indicated that galidesivir binds effectively to SARS-CoV-2 RdRp, suggesting its potential use to treat COVID-19.^290^

Cytidine analogs have also been investigated for COVID-19 treatment. One of the cytidine analogs, molnupiravir, is the synthetic ribonucleoside derivative N4-hydroxycytidine developed by Merck and Ridgebace. It is a prodrug of β-D-N4-hydroxycytidine (EIDD-1931), which was originally developed for treating seasonal influenza.^291^ Unlike the abovementioned remdesivir, which terminates the elongation of viral genes, molnupiravir contains two forms of tautomers that can pair with A and T,^35^ thus causing large mutations in RNA products and preventing SARS-CoV-2 replication. According to the results reported by Sheahan et al., administration of molnupiravir improved pulmonary function and reduced virus titer and weight loss in mice infected with SARS-CoV-2.^292^ On November 4, 2021, it was first approved by the UK Medicines and Health Products Regulatory Agency (MHRA) for treating adults with mild to moderate COVID-19. Thus, molnupiravir was the word’s first orally administered anti-SARS-CoV-2 drug. A recent study revealed that the SARS-CoV-2 Omicron variant is highly sensitive to molnupiravir.^293^ However, the potential side effect of molnupiravir of eliciting mutation in mammalian cells has raised concern.^294^ Azvudine is a cytosine analog which was also found to be efficient to treat SARS-CoV-2.^295^ It was previously approved for HIV inhibition.^296^ Recently, Zhang et al. observed that azvudine significantly inhibited viral load, promoted lymphocyte subsets, protected histological structures, and reduced inflammation caused by SARS-CoV-2 infection.^297^

Several guanosine analogs were reported to be efficient for inhibiting SARS-CoV-2.^298^ The most investigated one is ribavirin. It is a broad-spectrum antiviral drug with triazole structure, whose conformation is similar to that of guanosine.^299^ In 1970, it was first synthesized by Joseph T. Witkowski of ICN Pharmaceuticals.^300^ In 2013, it was approved by the FDA for the treatment of chronic HCV infection.^61^ Eslami et al. showed that combination therapy with ribavirin can effectively improve disease symptoms in severe COVID-19 patients.^301^ Later, results of an open-label randomized phase II trial showed that this triple therapy in hospitalized patients with COVID-19 pneumonia can effectively alleviate symptoms and shorten the duration of viral shedding and hospital stay in patients.^302^ Combination treatment with ribavirin, which is currently clinically available and cheap, with other antiviral drugs may become the treatment of choice in COVID-19 patients. In addition to ribavirin, the guanosine analog thioguanosine potentially inhibits SARS-CoV-2 by binding to M^pro^.^303^ The guanine analog triazavirin was reported to be a promising agent to treat SARS-CoV-2.^304^ A pilot trial by Wu et al. indicated that triazavirin can inhibit the tendency to bind to ACE2, and triazavirin showed a significantly better therapeutic effect and higher safety in the treatment of COVID-19 compared with a placebo or standard therapy.^305^

The uridylate analog sofosbuvir was also believed to play an anti-SARS-CoV-2 role.^306^ It was discovered in 2007 by Pharmasset (Gilead) and approved for HCV treatment.^307^ Previous studies have also shown that it can inhibit Zika virus replication.^308,309^ Sofosbuvir needs to be triphosphorylated to its active form (2’-F, Me-UTP) to be recognized by HCV polymerase, thereby preventing viral replication.^310^ A study by Chien et al. showed that the activated triphosphate form of Sofosbuvir can bind to RdRp of SARS-CoV-2.^311^ Currently, several clinical trials studying the effects of sofosbuvir on SARS-CoV-2 are being carried out.^301^ According to a multicenter Egyptian study involving 174 patients with COVID-19, patients receiving combination treatment with sofosbuvir/daclatasvir demonstrated shorter hospital stay, faster PCR negativity, and possibly reduced mortality.^312^ However, according to a meta-analysis by Kow et al., sofosbuvir-based direct-acting antiviral agents have no protective effects against the development of severe illness in patients with COVID-19 with the current dosing regimen.^313^ In a previous study, sofosbuvir demonstrated higher anti-viral efficiency against West Nile virus in hepatic cells than in lung cells.^314^ This liver-targeting characteristic of sofosbuvir raises concerns for its use in treating SARS-CoV-2. In this regard, future studies should be conducted to improve sofosbuvir’s targeting of the SARS-CoV-2-attacked organs by structural optimization or formulation improvement.

Favipiravir, a pyrazine analog with no nucleoside-like structure, can also be phosphorylated and acts as a nucleotide analog that selectively inhibits viral RdRp.^315^ It is being developed and manufactured by Toyama Chemical (a subsidiary of Fujifilm) and was approved for influenza virus treatment in Japan in 2014. An in vitro study showed that favipiravir exerts beneficial effects in Vero E6 cells infected with SARS-CoV-2 with a half-maximal effective concentration (EC_50_) of 61.88 μM and a half-cytotoxic concentration (CC_50_) of >400 μM.^276^ Many clinical trials proposed to use favipiravir in the treatment of COVID-19. Cai et al. reported that after favipiravir treatment, a significant improvement in chest CT of COVID-19 patients was observed, indicating that favipiravir is associated with better therapeutic responses in COVID-19 patients in terms of disease progression and viral clearance.^316^ In a multicenter randomized study, Dabbous et al. discovered that the patients who received favipiravir had a lower mean duration of hospitalization than patients in the chloroquine group.^317^ Thus, favipiravir has been recommended by Thailand’s Department of Disease Control for mild to moderate COVID-19 cases in both adults and children, while recommendations from India include mild COVID-19 patients with or without comorbidities.^45^ Furthermore, Rabie discovered a derivative of favipiravir named cyanorona-20 as a promising anti-SARS-CoV-2 compound.^318^ Pyrazine derivatives may serve as guides for further discovery of anti-SARS-CoV-2 agents.

As the basis of nucleotides, pyrimidines widely participate in viral metabolism. Thus, nucleobase analogs were found to effectively inhibit SARS-CoV-2 by various pathways. Among them, baricitinib, a pyrrolopyrimidine analog, is widely applied for treatment of severe COVID-19 in combination with remdesivir.^319^ Baricitinib is an oral selective inhibitor of JAK1 and JAK2.^320^ It was initially predicted by artificial intelligence algorithms as a potential treatment strategy against SARS-CoV-2. According to a study by Bronte et al., baricitinib improved the clinical outcomes of SARS-CoV-2 infection, affected the immune landscape in participants with COVID-19, and modified immune-suppressive features of myeloid cells.^321^ A study by Marconi et al. suggested that baricitinib reduces 28-day and 60-day mortality when used in addition to the current standard of care.^322^ As such, baricitinib plus standard of care could be a treatment option to reduce overall deaths globally. Another pyrrolopyrimidine analog, abivertinib, was found to depress cytokine production in patients with COVID-19.^323^ Several pyrimidine analogs have also been found to combat SARS-CoV-2. For example, according to a recent study conducted by Huntington et al., GLPG-0187, which bears a pyrimidin ring, effectively blocked SARS-CoV-2 pseudovirus infection across multiple viral variants, especially the Omicron and Delta pseudovirus variants, in a dose-dependent manner.^324^ Indu et al. reported that raltegravir combats SARS-CoV-2, because it demonstrated the highest interaction energy with M^pro^ and had high bioavailability among 65 FDA-approved small molecule antiviral drugs.^325^ Fostamatinib might be used to treat severe COVID-19.^326^ Other pyrimidine analogs, including ambrisentan and apilimod, were also reported to be promising agents for SARS-CoV-2 treatment.^327,328^

Another compound class, dsRNA, was also found to inhibit SARS-CoV-2. Rintatolimod, a Toll-like receptor 3 (TLR3) agonist, was reported to exert antiviral effects in human pancreatic cancer cells by activating the innate immune system, suggesting it could be used in the treatment of cancer patients who suffer from SARS-CoV-2 infection.^329^ Poly-ICLC is a synthetic complex of carboxymethylcellulose, polyinosinic-polycytidylic acid, and poly-L-lysine dsRNA.^330^ A phase I trial to study the safety and immunogenicity of poly-ICIC in healthy vaccinated COVID-19 adults is in its recruitment stage.

### Flavonoids

Flavonoids are a class of bioactive substances derived from plants. Chemically, flavonoids have a C6-C3-C6 skeleton structure, which consists of two phenyl rings and an oxygen heterocyclic ring.^331^ By regulating key enzymes participating in biological processes, flavonoids possess antioxidant, anticancer, anti-inflammatory, and antiviral properties.^332^ Due to their broad bioactivity, they may play complex roles to treat SARS-CoV-2 infection by blocking ACE2 receptor in host cells, directly inhibiting viral RdRp and M^pro^, and affecting the activity of various inflammatory enzymes (such as phospholipase A2, cyclooxygenases [COXs], TK, and so on).^333^ These mechanisms make flavonoids an excellent supportive care strategy for patients suffering from chronic post-COVID-19 syndrome.

Flavonols, also named 3-hydroxyflavones, are the most abundant and widely distributed flavonoids in the nature. Chemically, these molecules differ from many other flavonoids due to the hydroxyl group at position 3 of the flavonol skeleton. Quercetin, the most abundant flavonoids in edible plants, is a flavonol with five hydroxy groups placed at the 3-, 3′-, 4′-, 5-, and 7-positions. It has broad-spectrum antiviral ability against a variety of viruses, including HIV, poliovirus, Sindbis virus, respiratory viruses, Mayarovirus, and Mengo virus.^334,335^ Pan et al. reported that quercetin may exert anti-SARS-CoV-2 effects by affecting the binding of viral S protein to the ACE2 receptor.^336^ Further, the anti-SARS-CoV-2 effect of quercetin was also thought to be achieved by (i) inhibiting M^pro^ and PL^pro^ proteinase of SARS-CoV-2 and (ii) acting as a zinc ionophore.^337^ Currently, several clinical studies of quercetin are underway. A phase IV clinical study supported by the Ministry of Health of Saudi Arabia on quadruple therapy with quercetin, zinc, bromelain, and vitamin C for COVID-19 patients is in its recruitment stage (NCT04468139). Myricetin, a 7-hydroxyflavonol, has been isolated from the leaves of *Myrica rubra* and other plants. In research conducted by Su et al., myricetin inhibited M^pro^ at >90% at a concentration of 10 μM, and its EC_50_ value in Vero E6 cells infected with SARS-CoV-2 was 8.00 μM.^94^ The 3-hydroxyl group of flavonol can be glycosylated, thus forming flavonol glycosides, which are found in plants. As a quercetin O-glycoside, quercitrin is obtained by placing an alpha-L-rhamnosyl moiety at position 3 of quercetin via a glycosidic linkage. Several in silico studies have reported that quercitrin may be used against SARS-CoV-2 based on its affinity to the serine protease TMPRSS2, M^pro^, and PL^pro^.^338–340^

There are a series of compounds whose backbone consists of a flavonol structure. They have also been found to be effective in combating COVID-19. For example, flavonolignans are a family of compounds containing a flavonol moiety linked together with coniferyl alcohol.^341^ Silymarin, extracted from the botanical source *Silybum marianum*, is a mixture of flavonolignans (silybin, isosilybin, silychristin, and siliandrin) and a flavonol (taxifolin).^342^ It is commonly known for its hepatoprotective potential.^343^ Its anti-SARS-CoV-2 effect was thought to be achieved by inhibiting the expression of the host cell surface receptor TMPRSS2.^342^ Hanafy et al. developed silymarin/curcumin dual-loaded BSA nanoparticles as an inhalable delivery system to treat pneumonia.^344^ According to their results, silymarin exhibited antiviral activity against SARS-CoV-2 at a concentration of 25 μg/mL in vitro. They reported that silymarin could protect the lungs during SARS-CoV-2 infection due to their anti-inflammatory and antioxidant effects, and it could inhibit the ACE2 receptor, thus preventing viral entry. As a natural-derived compound mixture, silymarin might be a good option for treating COVID-19 owing to its multifunction properties. A phase III clinical study of silymarin is in its recruitment stage, which is aimed at assessing the clinical outcome in adults with COVID-19 pneumonia under standard care plus placebo or oral silymarin (NCT04394208).

In addition to the abovementioned flavanols, the anti-SARS-CoV-2 effects of flavones, which have a 2-phenyl-1-benzopyran-4-one backbone, were also studied. Luteolin is the most investigated flavone compound. Luteolin is a flavone which bears four hydroxy groups located at the 3′-, 4′-, 5-, and 7-locations. It is obtained from the plant *Reseda luteola*. It was first isolated in pure form and named in 1829 by the French chemist Michel Eugène Chevreul.^345^ Results obtained from relaxed complex scheme analysis, classical molecular docking simulations, and metadynamics simulations suggest luteolin blocks SARS-CoV-2 entry into cells.^346,347^ A system pharmacology and bioinformatic analysis study conducted by Xie et al. indicated it has great potential to be used for treating COVID-19/asthma comorbidity due to its effects on viruses, regulating inflammation and immune responses, reducing oxidative stress, and regulating blood circulation.^348^ Luteolin was found to be safe for human use and showed good drug properties. Clinical results suggest that oral luteolin supplementation improves the recovery of olfactory function after COVID-19. Besides the above common flavones, amentoflavone, a hydroxyflavone and bioflavonoid, also has shown binding affinity with M^pro^, RdRp, NSP13, NSP15, and ACE2 in several in silico surveys.^349–351^ Similar to flavanols, the hydroxy groups of flavones can be glycosylated, thus forming flavone glycosides. Baicalin, a 7-O-glucuronide of baicalein, is a biologically active flavonoid of natural origin obtained primarily from the roots of *Scutellaria baicalensis* Georgi. Zandi et al. have demonstrated that baicalein and its aglycon baicalein can directly inhibit the activity of SARS-CoV-2 RdRp and that it exhibits in vitro anti-SARS-CoV-2 activity with an EC_50_ of 4.5 µM and an EC_90_ of 7.6 µM.^352^ Su et al. also found its binding activity with M^pro^ and proved its anti-SARS-CoV-2 activity in vitro. Their further study revealed that baicalin and baicalein as two bioactive ingredients of Shuanghuanglian (a Chinese traditional medicine) provides supporting evidence for the antiviral activity of Shuanghuanglian. However, their exact antiviral ability has to be verified in animal models or clinical trials.

The effects of flavanols represented by epigallocatechin gallate (EGCG) against COVID-19 have also been studied. EGCG is a phenolic antioxidant found in a number of plants, including green and black tea, with reported antiviral effects against influenza virus, HIV, and HBV.^353,354^ Unlike other flavonoids with a chromone part, it bears a 2-phenyl-3,4-dihydro-2H-chromen-3-ol skeleton.^355^ EGCG exerts inhibitory effects on SARS-CoV-2 replication through its actions on ACE2, M^pro^, and RdRp.^356^ Jang et al. demonstrated that EGCG inhibits SARS-CoV-2 M^pro^ activity in 293T cells in a dose-dependent manner without signs of cytotoxicity at any dose used.^357^ Chiou et al. conducted an in vitro study on the inhibitory effects of EGCG against SARS-CoV-2 M^pro^. EGCG inhibited the activity of SARS-CoV-2 M^pro^, thus suggesting its potential application in the treatment of SARS-CoV-2 infection.^358^ It is worth noting that a clinical phase II/III study of EGCG is underway to determine its chemoprophylactic effects on COVID-19 in healthy workers (NCT04446065). Other flavanols, including cianidanol,^359^ epicatechin gallate,^360,361^ and procyanidin,^362^ have also been found to have potential anti-SARS-CoV-2 effects in vitro.

### Phenylpropanoids

Phenylpropanoids are a family of plant-derived compounds with a C6–C3 structure. In general, phenylpropanoids are derived from the shikimic acid pathway via phenylalanine and tyrosine. This phenylpropanoid metabolism pathway is a major anabolic pathway in plants, which plays a vital role in several processes, especially biotic and abiotic stress responses.^363^ Phenylpropanoids act as antioxidants and free radical scavengers. Their applications as antioxidant, anticancer, antiviral, anti-inflammatory, and antibacterial agents have attracted interest.^364^ Several phenylpropanoids were found to exert anti-SARS-CoV-2 effects. Some of them have demonstrated potential anti-SARS-CoV-2 effects in vitro or by computational analysis.

Hydroxycinnamic acid derivatives belong to the basic phenylpropanoids. Based on the C6-C3 structure, they also possess an aromatic carboxylic acid substituted by phenolic hydroxyl groups. As a common derivative of hydroxycinnamic acid, caffeic acid possesses a phenyl ring substituted by hydroxy groups at the 3- and 4-positions.^365^ It is an orally bioavailable small molecule mainly found in *Pavetta indica* and *Eupatorium cannabinum*. Further studies have shown its potential antiviral activity against HBV^366^ and HPIV3.^367^ Several in silico molecular docking studies have revealed it could specifically bind to SARS-CoV-2 M^pro^^368^ and Membrane protein.^369^ Chlorogenic acid, the ester of caffeic acid and quinic acid, is often found in coffee and black tea. Several studies have pointed out that chlorogenic acid and its derivatives have good antiviral activity against various types of viruses, including HIV, influenza A virus, herpes simplex virus (HSV), and hepatitis B virus (HBV).^370^ Its anti-SARS-CoV-2 ability was first predicted by Yu et al., whose molecular docking study revealed that chlorogenic acid could stably bind with ACE2, indicating it may inhibit SARS-CoV-2 entry into cells.^371^ Another molecular docking simulation conducted by Gizawy et al. suggested that chlorogenic acid can interact with the Asn-142, His-164, Arg-188, and Met-165 residues of the active site in M^pro^ of SARS-CoV-2.^372^ According to their in vitro study on Vero E6 cells, chlorogenic acid had an IC_50_ of 360 μg/mL and a selective index (CC_50_/IC_50_) of 8 against SARS-CoV-2. Chen et al. found that chlorogenic acid and its isomers (chlorogenic acid, neochlorogenic acid, and cryptochlorogenic acid) all exhibited ACE2 inhibitory activities with IC_50_ values of about 40 μM.^373^ As a chlorogenic acid derivative, isochlorogenic acid A is the diester obtained by the condensation of the hydroxy groups at positions 3 and 5 of (−)-quinic acid with the carboxy group of trans-caffeic acid. Recent computational studies have predicted it to have binding ability with M^pro^ of SARS-CoV-2.^374,375^ Salvianolic acid B (Sal-B), one of the main active ingredients of *Salvia miltiorrhiza*, is a hydroxycinnamic acid with strong antioxidant effects.^376^ Hu et al. revealed that by binding to the RBD of S protein and ACE2, Sal-B can inhibit the entry of SARS-CoV-2 pseudovirus into cells that highly express ACE2.^377^ A similar anti-SARS-CoV-2 effect can be also achieved by Sal-A and Sal-C. According to a study by Wang et al., Sal-A dose-dependently alleviates the pathological alterations in mice with acute lung inflammation due to infection with SARS-CoV-2 S protein-pseudotyped virus in a dose-dependent manner.^378^ Sal-C has been reported to potently bind to the 6-HB core of S protein, thereby inhibiting SARS-CoV-2 infection.^379^ According to the in vitro results, Sal-C potently inhibits the membrane fusion of S protein–overexpressing HEK293T and Vero E6 cells with an IC50 of 1.71 μM.

Like other natural products, hydroxycinnamic acid derivatives often occur as glycosides in nature. For example, forsythoside A is a phenylethanol glycoside product isolated from the dried fruit of forsythia, of which it is the main active ingredient.^380,381^ Chen et al. demonstrated that forsythoside A acid could form suitable steric complementarities with the binding interface of ACE2 with SARS-CoV-2 S protein by ACE2 bio-chromatography screening.^373^ Fu et al. found it has strong docking affinities with S protein’s RBD of SARS-CoV-2 and its variants (Alpha [B.1.1.7], Beta [B.1.351], and Delta [B.1.617]), as well as NRP1 and M^pro^ .^382^ Moreover, biolayer interferometry binding (BLI) analysis results revealed that forsythoside A may block or interfere with the binding of the RBD to other receptors in the body (e.g., ACE2) by binding to the RBD.^383^

Besides hydroxycinnamic acid derivatives, coumarins and lignans also belong to the phenylpropanoids. Coumarins bear a 2H-chromen-2-one (2H-1-benzopyran-2-one or benzo-alpha-pyrone) ring.^384^ Bergamottin, a natural product found in bergamot, exhibits a structure similar to that of furanocoumarin. Zhou et al. have reported its inhibitory activity against SARS-CoV-2 both in vitro and in vivo.^385^ According to their results, bergamottin interferes with various stages of viral life cycle, including blocking the viral fusion and reducing the viral RNA replication, and effectively protects a golden Syrian hamster model from SARS-CoV-2 infection. It is worth noting that bergamottin inhibits CYP450 activity like ritonavir, which means that it may be promising to combine it with other anti–SARS-CoV-2 drugs. Lignans are constituted by the union of two phenylpropane units.^386^ As a lignan and glycoside, phillyrin is the main active ingredient of the traditional Chinese medicine *Forsythia suspensa*. Ma et al. reported that phillyrin could significantly inhibit SARS-CoV-2 and HCoV-229E replication in vitro.^387^ Further, Lai et al. discovered that phillyrin could be used to treat COVID-19 and influenza co-infection since it not only inhibits the replication of both viruses, but also possesses the ability to regulate hypoxia-cytokine storm based on bioinformatics network pharmacology analysis.^388^

### Terpenoids

Terpenoids are a large class of natural compounds based on isoprene units. They display various biological activities and have diverse structures. Their lipophilicity was assumed to empower their tendency to enter into cellular membranes, thus affecting functions of membrane proteins or disrupting membrane integrity. Terpenoids exert multiple effects, including anti-inflammatory and antiviral effects.^389^ By enhancing the adaptive immune response and inverting the chronic inflammatory response elicited by the virus, terpenoids are thought to assist in the treatment of COVID-19 and associated symptoms.^390,391^ Many preclinical studies have found terpenoids with direct anti-SARS-CoV-2 effects by binding them to proteins or viral receptors.

Sesquiterpenoids are a class of enormously diverse natural products derived from a 15-carbon precursor. Artemisinin is the sesquiterpene endoperoxide lactone extracted from the herb *Artemisia annua* as the basis for the currently preferred treatment for malaria.^392,393^ Antiviral activities of artemisinin and its analogs against HSV type 1, Epstein–Barr virus, HBV, HCV, bovine viral diarrhea virus, and human cytomegalovirus have been reported.^394–396^ Molecular dynamics analysis revealed that artemisinin interacts with Lys-353 and Lys-31, which are binding hotspots of the SARS-CoV-2 S protein, in two patterns.^397^ Cao et al. conducted an in vitro study of artemisinin analogs, which revealed Arteannuin B exerts the strongest anti-SARS-CoV-2 effects, with an EC_50_ of 10.28 ± 1.12 μM.^398^ Artesunate and dihydroartemisinin showed similar EC_50_ values of 12.98 ± 5.30 μM and 13.31 ± 1.24 μM, respectively. Another in vitro study found that artemisinin alone showed an estimated IC_50_ of about 70 μM, and the clinically used artemisinin derivatives artesunate, artemether, and dihydroartemisinin were ineffective or cytotoxic at elevated micromolar concentrations.^399^ An open-label, non-randomized controlled trial by Li et al. revealed that the combination of artemisinin and piperaquine shortens the time SARS-CoV-2 remains in the body.^400^ Besides artemisinin analogs, other sesquiterpenoids, including beta-eudesmol,^401^ nootkatone,^402,403^ and lactupicrin,^404^ were all included in computational studies of SARS-CoV-2.

Diterpenoids are a chemically heterogenous group of compounds, all with a C20 carbon skeleton based on four isoprene units.^405^ Andrographolide is a diterpenoid extracted from traditional Chinese medicine. It is a well-known diterpenoid with broad therapeutical applications, including the inhibitory effect on HIV virus, influenza A virus H1N1, H3N2 and influenza B virus.^406^ In a molecular docking study of Rajagopal et al., andrographolide was predicted to bind to M^pro^ of SARS-CoV-2. Further, according to an in vitro anti-SARS-CoV-2 assay by Hu et al., andrographolide (EC_50_ = 11.12 µM, CC_50_ = 95.73 µM, SI = 8.61) showed excellent anti-SARS-CoV-2 activity.^391^ A phase III clinical study using andrographolide for COVID-19 treatment is in the recruitment stage in Thailand (NCT05019326). Another diterpenoid, paclitaxel, is a compound extracted from the Pacific yew tree *Taxus brevifolia* with antineoplastic activity. Paclitaxel binds to tubulin and inhibits the disassembly of microtubules, thereby resulting in the inhibition of cell division. This agent also induces apoptosis by blocking the function of the B-cell Leukemia 2 (Bcl-2) protein, which inhibits apoptosis. Using a network-based drug repurposing strategy, Adhami et al. found that paclitaxel has four interactions with genes associated with SARS-CoV-2 infection, which is the most remarkably identified candidate drug for COVID-19.^407^ Molecular docking and molecular dynamics simulation analyses by Pingali et al. indicated that paclitaxel has high affinity to RdRp of SARS-CoV-2.^408^ However, the impairment of the helper/suppressor T cell ratio and depletion of CD4 + T cells, CD8 + T cells, and natural killer cells during paclitaxel therapy can result in susceptibility to infection and pneumonia.^409^ Paclitaxel also increases the alveolar capillary membrane permeability, resulting in several adverse effects, including pulmonary diffusion dysfunction, which raises concerns for COVID-19 treatment.^410^ Other diterpenoids, including triptolide,^411^ oridonin,^412,413^ and carnosic acid,^414,415^ were also identified to have anti-SARS-CoV-2 activities by computational methods.

The beneficial effects of triterpenoids such as glycyrrhizic acid in COVID-19 were investigated. Glycyrrhizic acid is obtained from licorice, and it has been shown to inhibit various viruses, such as HIV, HBV, and herpes zoster virus.^416^ Molecular docking studies have identified its binding affinity with M^pro^, S protein, and NSP15 of SARS-CoV-2. Zhao et al. developed highly biocompatible glycyrrhizic acid nanoparticles with the ability to inhibit murine coronavirus MHV-A59.^417^ Their results indicated that glycyrrhizic acid nanoparticles could reduce proinflammatory cytokine production caused by MHV-A59 or SARS-CoV-2 N protein, indicating their potential for COVID-19 treatment. Bardoxolone and bardoxolone methyl are oleanolic acid-derived synthetic triterpenoid compounds that activate the Nrf2 pathway and inhibit the NF-κB pathway, and they can be used to treat chronic kidney diseases.^418^ Sun et al. determined the binding activities of both compounds to the active site cysteine of SARS-CoV-2 M^pro^ with computational analyses.^419^ Further in vitro experiments showed that bardoxolone methyl and bardoxolone inhibit SARS-CoV-2 replication in Vero E6 cells with EC_50_ values of 0.29 μM (SI = 23.9) and 0.43 μM (SI = 56.6), respectively, and in human Calu-3 cells with EC_50_ values of 0.20 μM (SI = 5.8) and 0.42 μM (SI = 28.2), respectively. Alpha-hederin is a triterpenoid saponin that is produced by attaching a 2-O-(6-deoxy-alpha-L-mannopyranosyl)-alpha-L-arabinopyranosyl residue to hederagenin at position 3 via a glycosidic linkage. Studies have shown its potential to inhibit SARS-CoV-2 RdRp, M^pro^, and the S protein RBD domain by molecular docking methods.^420,421^

Carotenoids are a group of compounds with a polyene chain backbone, mostly eight-isoprenoid building blocks (tetraterpenoids). They are biosynthesized by plants, bacteria, and fungi but not humans; therefore, humans need to obtain them from the diet.^422^ Carotenoids exhibit many health and pharmaceutical effects in the body, and they have been used to treat COVID-19 and related symptoms.^423^ The carotenoid crocetin is a 20-carbon natural carotenoid which is also a diterpenoid and a vitamin A analog.^424^ Kordzadeh et al. identified it as a candidate drug for COVID-19 treatment based on its high binding energies to S protein and M^pro^ of SARS-CoV-2 virus.^425^ Further, in a phase I/II clinical trial on COVID-19 patients suffering from severe respiratory complications, a single injection of LEAF-4L6715 (a liposomal nanocarrier encapsulating crocetin) enhances the oxygenation of vascular tissue and therefore has the potential to improve the clinical outcomes of ARDS and COVID-19 in severely impacted patients.^426^ The sodium salt of the trans-isomer of crocetin, trans sodium crocetinate, also has entered a phase I/II clinical trial for treatment of COVID-19 patients. Crocin is a glucoside derived from crocetin. As an antioxidant, crocin has been investigated for the treatment of hyperglycemia, metabolic syndrome, hypertriglyceridemia, and hypercholesterolemia.^427,428^ It was reported that crocin has the potential to limit the progression and severity of SARS-CoV-2 infection due to its antioxidant, anti-inflammatory, and immunomodulatory properties.^429^ By employing computational methods, Kordzadeh et al. and Aanouz et al. identified its binding affinity towards the M^pro^ of SARS-CoV-2.^401,425^ Stalin et al. also reported its distinctive strong interaction with the RBD of SARS-CoV-2 S protein.^430^ Beta-carotene is a vitamin A precursor composed of two retinyl groups. In critical COVID-19 patients, the concentration of beta-carotene is decreased compared to the reference range.^431^ In a study conducted by Xia et al., the binding affinity of beta-carotene to the AKT1 pocket was determined, suggesting potential therapeutic effects on COVID-19.^432^ Astaxanthin is derived from a hydride of beta-carotene. It is a carotenoid with no vitamin A activity but still has antioxidant and anti-inflammatory properties. Some studies have shown that it can be used to prevent and counteract the symptoms of COVID-19.^433,434^ An in -silico study also revealed that it can interact with SARS-CoV-2 proteins (M^pro^, RdRp, NSP15, and S protein).^435^ In addition, the three main forms of vitamin A, retinol, retinal, and tretinoin, are also carotenoids with potential anti-COVID-19 effects. Vitamin A is the most evaluated nutrient due to its impact on immunity. It is a key regulator of immune function and augments the innate response to RNA viruses. The dsRNA formed within the cells by viral pathogens is primarily sensed by pattern recognition receptors including retinoic acid-inducible gene I (RIG-I) and RIG-I-like receptors (RLRs). Vitamin A has been demonstrated to decrease mortality due to measles and Ebola in clinical studies.^436,437^ Based on this, vitamin A is considered to hold benefits for COVID-19 patients as therapeutic agent or as adjuvant with vaccines. Although with unspecific antiviral mechanisms and effects, it is encouraging to study its benefit for COVID-19 patients due to its high safety, low cost, and availability in most of the developing countries. To verify its function, two phase II clinical trials have been registered to evaluate the effects of vitamin A supplementation on disease in children with COVID-19 (NCT04920760) or in patients with COVID-19-related olfactory dysfunction (NCT04900415).

Cannabinoids are a diverse group of compounds derived from *Cannabis sativa*. Most of them are terpenoids with complex anti-inflammatory and antiviral effects.^438^ The effects of cannabinoids on COVID-19 patients have been investigated.^439^ Cannabidiol is an orally available cannabinoid that is largely related to the human endocannabinoid system. It has been approved by the FDA and EMA for treatment of Dravet syndrome and Lennox–Gastaut syndrome.^440–442^ Raj et al. screened 32 cannabinoids with binding affinity to SARS-CoV-2 M^pro^.^443^ Five cannabinoids were selected, and their antiviral abilities were tested in vitro. Cannabidiol (IC_50_ = 7.91 μM) was found to be exert more potent antiviral effects against SARS-CoV-2 in vitro compared to the reference drugs lopinavir, chloroquine, and remdesivir (IC_50_ ranges of 8.16–13.15 μM). Nguyen et al. reported that cannabidiol treatment could significantly inhibit SARS-CoV-2 replication in mice.^444^ Moreover, they found that patients with a medical record of cannabidiol for seizure-related conditions exhibited a lower SARS-CoV-2 infection rate than non-cannabidiol patients, which revealed cannabidiol is negatively associated with indications of SARS-CoV-2 infection. Currently, seven clinical trials on cannabidiol for the treatment of COVID-19 and related diseases are underway. Based on these studies, cannabidiol may be a promising drug for treating COVID-19. Dronabinol, also named delta-9-tetrahydrocannabinol (Δ^9^-THC), is the primary psychoactive component of cannabis (marijuana). Mohammed et al. demonstrated that Δ^9^-THC could lead to a 100% survival rate, decreased lung inflammation, and the suppression of cytokine storm in a mouse model of ARDS induced by staphylococcal enterotoxin B, suggesting Δ^9^-THC could be used to treat ARDS associated with COVID-19.^445^ Pitakbut et al. reported that Δ^9^-THC acts as an inhibitor against both M^pro^ and ACE2 with IC_50_ values of 16.23 ± 1.71 µM and 11.47 ± 3.60 µM, respectively.^446^

### Steroids

Steroids are compounds that contain four cycloalkane rings with a perhydrocyclopentano[α]phenanthrene core structure. They are found in plants and animals. Synthetic steroids were developed to enhance their biological activities. Steroids play an important role in people’s lives.^447^ Various sex hormones, corticosteroids, vitamin D, cholesterol, and cardiac glycosides are natural steroid compounds with vital physiological activity. Steroids have been used by the pharmaceutical industry and have various applications, such as anticancer drugs, anti-inflammatory agents, anticonvulsants, contraceptives, anti-autoimmune disease drugs, and fertility stimulants.^448,449^ Steroids such as glucocorticoids exert effects on severe COVID-19 patients due to their anti-inflammatory effects. Other steroids, including vitamin D and sex hormones, are also beneficial for COVID-19 patients due to their immunomodulatory action.^450–452^

The effectiveness of two main classes of corticosteroids, glucocorticoids and mineralocorticoids, in COVID-19 treatment were explored in different observational studies.^453^ During the initial phase of the SARS-CoV-2 pandemic, 44.9% of hospitalized patients with COVID-19 pneumonia received glucocorticoid therapy.^454^ The clinical practice guideline of “The Infectious Diseases Society of America” recommends the use of glucocorticoids in severe COVID-19 patients.^455^ As a synthetic glucocorticoid, dexamethasone is derived from a hydride of a pregnane with anti-inflammatory function. In a preliminary report of a controlled, open-label trial comparing a range of possible treatments for hospitalized patients with COVID-19, dexamethasone treatment showed a reduction in 28-day mortality in patients with COVID-19 who received respiratory support.^456^ Another randomized clinical trial comparing intravenous dexamethasone plus standard care with standard care revealed a statistically significant increase in the number of days alive and the number of days free of mechanical ventilation over a 28-day period in the dexamethasone group.^457^ The glucocorticoid methylprednisolone is an FDA-approved anti-inflammatory and systemic immunosuppressive corticosteroid. A study proposed that high-dose methylprednisolone significantly decreased the recovery time compared with dexamethasone in COVID-19 patients.^458^ Through describing the clinical characteristics and outcomes in patients with COVID-19 pneumonia who developed ARDS or died, Wu et al. found that treatment with methylprednisolone decreased the risk of death among patients with ARDS.^459^ In another clinical trial on 46 severe COVID-19 patients, Wang et al. concluded that early, low-dose, and short-term application of methylprednisolone was associated with better clinical outcomes, which revealed it should be considered before the occurrence of ARDS.^460^ However, there are concerns since Li et al. reported that high-dose methylprednisolone potentially increased the mortality of patients with severe COVID-19.^461^ The possible reasons might be the delayed clearance of virus under high-dose glucocorticoid treatment. Thus, despite the effective anti-inflammatory effect, glucocorticoids should be applied carefully, giving due consideration to factors such as initiation of the therapy, dosage, and route of administration.

Vitamin D is a group of steroids that have an open ring structure. It is an essential metabolite clinically associated with infection, reproduction, the cardiovascular condition, and cancer.^462–466^ In a retrospective, observational study of Carpagnano et al., COVID-19 patients with severe vitamin D deficiency had a significantly higher mortality risk and poor prognosis rate, suggesting adjunctive treatment with vitamin D might improve disease outcomes.^467^ The proposed mechanisms whereby vitamin D reduces the risk of COVID-19 have been clearly summarized in a review of Barrea et al..^468^ For these reasons, numerous clinical studies have used vitamin D as supplementation in COVID-19 treatment. Cholecalciferol, also known as vitamin D3, is the endogenous form of vitamin D. According to a randomized clinical trial in patients with mild to moderate COVID-19, 5000 IU daily oral vitamin D3 supplementation for 2 weeks reduces the time to recovery for cough and gustatory sensory loss among patients.^469^ Calcifediol is the major circulating metabolite of vitamin D3, and it is the best indicator of the body’s vitamin D stores. In a pilot randomized clinical study in 76 patients hospitalized with COVID-19 infection, administration of a high dose of calcifediol significantly reduced the need for ICU admission of patients when all of them received the best available therapy and the same standard care.^470^ A retrospective, multicenter, open, non-randomized cohort study reported by Alcali-Diaz et al. evaluated if calcifediol supplementation influences in-hospital mortality of COVID-19 patients under standard care and the best available treatment.^471^ According to their results, treatment with calcifediol was significantly associated with lower in-hospital mortality during the first 30 days. Moreover, a multicenter, randomized, double-blind, placebo-controlled clinical trial revealed that calcifediol was able to improve the immune function of COVID-19 patients by increasing blood lymphocyte counts.^472^

Sex hormones are another class of steroids widely studied for COVID-19 treatment. Adverse outcomes are more common among elderly and male COVID-19 patients. The levels of sex hormones such as progesterone, which has been shown to modulate a more robust immune response, are low in these people.^473,474^ Based on these observations, it is rational to consider sex hormones for treatment to alleviate COVID-19 inflammatory and cytokine storm events, as they can influence immune system function against SARS-CoV-2 infection, thus reducing the adverse effects of COVID-19. Estradiol, a 17-β-hydroxy steroid, is a naturally occurring hormone in females. Studies revealed that it may combat COVID-19 by inhibiting the SARS-CoV-2 S-protein-induced ACE2-dependent activation of NOX2, MCP-1, and ROS production.^475,476^ Baristaite et al. reported that treatment of A549 human lung epithelial cells with 17-β-estradiol reduced the cellular mRNA levels of ACE2 and TMPRSS2.^477^ This outcome may contribute to reduced SARS-CoV-2 infection of lung epithelial cells. Estrogen is thought to inhibit initial viral responses and attenuate cytokine-storm-induced endothelial dysfunction, so it might serve as a novel therapy for COVID-19 patients.^476^ Progesterone is another sex hormone with immunomodulatory and anti-inflammatory functions. Su et al. revealed that higher levels of progesterone alleviate COVID-19 symptoms, since progesterone promotes the innate antiviral response both in vitro and in vivo.^478^ Yuan et al. indicated that treatment with progesterone ameliorated the severity of SARS-CoV-2-caused pneumonia in a Syrian hamster model.^479^ In addition, a randomized, controlled pilot trial suggested that supplementation with progesterone in hospitalized men with moderate to severe COVID-19 resulted in shorter periods of oxygen supplementation (median, 4.5 vs. 7.5 days) and shorter hospitalization periods (median, 7.0 vs. 9.5 days) as compared with control subjects.^480^

### Azoles

Azoles are nitrogen-, sulfur-, and oxygen-containing compounds with a five-membered ring system. Azoles comprise various rings, including thiazole, oxazole, triazole, imidazole, and pyrazole. Most of them are known as antifungal agents, and other bioactivities such as antidiabetic, immunosuppressant, anti-inflammatory, anticancer, and antiviral effects also contribute to their pharmaceutical functions.^481–483^ Many synthetic small molecules bearing core structures of azoles have been studied in the fight against COVID-19.

Thiazoles are five-membered heterocyclic compounds containing sulfur and nitrogen. Nitazoxanide belongs to the class of thiazoles, and is also a synthetic benzamide.^484^ As a broad-spectrum antiviral drug, nitazoxanide inhibits a broad range of influenza A and B viruses including influenza A (pH1N1) and the avian A (H7N9) virus, as well as viruses that are resistant to neuraminidase inhibitors.^485^ Riccio et al. demonstrated that nitazoxanide could hamper the glycosylation of SARS-CoV-2 S protein, thus hindering infectivity of the virus.^486^ This study also revealed that nitazoxanide is equally effective against different variants of SARS-CoV-2, including the Delta variant. According to a preprint provided by Miorin et al., nitazoxanide exhibited an IC_50_ of 4.04 μM in Vero E6 cells against SARS-CoV-2, and a significant inhibitory effect was observed in different human cell lines including stem cell-derived human alveolar epithelial type 2 cells.^487^ This in vitro inhibitory effect was also confirmed against different SARS-CoV-2 variants (Beta, Gamma, and Delta). Moreover, this study also confirmed the antiviral activity of nitazoxanide by oral treatment in hamsters. The clinical application potential of nitazoxanide was also verified in placebo-controlled trial.^488,489^ The safety of high-dose nitazoxanide was also proved in a phase I clinical trial in healthy volunteers recently.

Oxazole is a five-membered heteroarene containing an oxygen in the 1-position and a nitrogen in the 3-position.^490^ Asapiprant, which contains an oxazole ring, is an antagonist of the prostaglandin D2 receptor (PTGDR).^491^ According to a study reported by Wong et al., treatment with asapiprant could protect aged mice from lethal SARS-CoV-2 infection, since elevated levels of prostaglandin D2 (PGD2) contribute to poor outcomes in SARS-CoV-2-infected aged mice.^492^ Proxalutamide is an androgen receptor antagonist, which also contains an oxazole ring, and it exhibited anti-SARS-CoV-2 potential in a clinical trial.^493^ Al-Wahaibi et al. synthesized novel oxazole-based macrocycles and evaluated their antiviral activities in vitro.^494^ Isopropyl triester-13 and triacid-14 exhibited IC_50_ values of 18.3 and 18.95 μM, respectively, on Vero E6 cells against SARS-CoV-2. Moreover, compound 13 exhibited a high inhibitory activity against M^pro^ of SARS-CoV-2 with an IC_50_ of 2.58 µM. Rivaroxaban, an orally bioavailable oxazolidine derivative, is an anticoagulant and a direct factor Xa inhibitor. Since COVID-19 can manifest with hypercoagulability, pulmonary intravascular coagulation, microangiopathy, and venous thromboembolism or arterial thrombosis, it is recommended to provide thromboprophylaxis with rivaroxaban in postdischarge patients.^495^

Triazole is a five-membered aromatic heterocyclic compound containing three nitrogen atoms. Itraconazole is a triazole antifungal agent used for treatment of systemic and superficial fungal infections.^496^ According to a study by Damme et al., itraconazole has antiviral activity in human Caco-2 cells with an EC_50_ of 2.3 μM against SARS-CoV-2.^497^ Yang et al. found that itraconazole could inhibit viral entry by targeting the 6-HB fusion core of SARS-CoV-2 S protein.^498^ In addition, Schloer et al. reported that traconazole–remdesivir combinations display synergistic effects and inhibit production of SARS-CoV-2 particles with >90% efficiency.^499^ According to their results, we can conclude that by interfering with different steps of the viral cycle, combination of drugs might be an effective and feasible way to combat fast-spreading SARS-CoV-2 variants. Selinexor contains structures of both triazole and hydrazine. It is a first-in-class small molecule inhibitor of chromosome region maintenance 1 protein (CRM1, also known as exportin 1 [XPO1]), with potential antineoplastic activity.^500–502^ By generating a series of transgenic fly lines for individual SARS-CoV-2 genes, Zhu et al. found that expression of ORF6 leads to reduced viability and tissue defects of flies, and selinexor could attenuate these phenotypes.^503^ Further experiments verified that ORF6 is a highly pathogenic protein encoded by the SARS-CoV-2 genome in human cell lines; thus, selinexor is a candidate drug for treatment of SARS-CoV-2-ORF6 protein-induced cellular damage.^504^ Kashyap et al. found that selinexor treatment reduced the viral load in the lungs and protected against tissue damage in the nasal turbinates and lungs in a ferret model of COVID-19.^505^ Bemcentinib is also a synthetic triazole with antifungal activity. It has an EC_50_ of 1.1 μM against SARS-CoV-2 in Vero E6 cells.^506^ Sitagliptin is a triazolopyrazine and a trifluorobenzene with multiple activities, including inhibitory effects on dipeptidyl peptidase-4 (DPP-4). DDP-4 is a target protein of the SARS-CoV-2 S protein; thus, sitagliptin is a candidate drug for COVID-19 treatment.^507^ Solertes et al. demonstrated that treatment with sitagliptin in hospitalized patients with type 2 diabetes and COVID-19 was associated with reduced mortality.^508^

The effects of other azoles, including pyrazole and selenzole, on COVID-19 were also studied. Ibrutinib is a pyrazolopyrimidine and a member of the acrylamides, and serves as an oral inhibitor of BTK that is used in the therapy of refractory chronic lymphocytic leukemia and mantle cell lymphoma.^509,510^ Treon et al. demonstrated that it may provide protection against lung injury and even improve pulmonary function in hypoxic patients with COVID-19.^234^ Five of the six COVID-19 patients receiving ibrutinib for Waldenstrom macroglobulinemia showed a steady improvement and resolution of COVID-19-related symptoms. Similar phenomena were observed in other reports of patients who have leukemia and COVID-19 at the same time. Ebselen is a benzoselenazole with anti-inflammatory, antioxidant, and cytoprotective activity.^511^ Jin et al. identified it as an antiviral agent targeting M^pro^ of SARS-CoV-2, and ebselen exerted inhibitory effects against SARS-CoV-2 with an EC_50_ of 4.67 μM.^68^ Two phase II clinical trials assaying ebselen’s effect in either moderate or severe COVID-19 patients are in the “enrolling by invitation” stage. Other compounds with an azole ring, such as zanubrutinib,^512^ acalabrutinib,^237^ and azilsartan,^513^ were also found to have affinity to SARS-CoV-2, and benzopyranylpyrazole-based hit compounds were demonstrated to inhibit SARS-CoV-2 replication in cells.^514^

### Amides

Amides are compounds derived from oxoacids by replacement of an acidic hydroxy group with an amino group or a substituted amino group. The amide group plays a vital function in the composition of many bioactive compounds, including amino acids, peptides, and small molecule drugs. Due to their ability to form hydrogen bonds inside pockets of target proteins, amides have gained increasing attention in drug design and development.^515,516^ Amides, especially peptidomimetics and derivatives of amino acids with binding affinity to host proteases or SARS-CoV-2, have been designed or repurposed in many studies.^41^

Derivatives of amino acids are amides with broad medicinal values and development prospects. They were also widely investigated in recent COVID-19 research, especially as inhibitors of M^pro^. Paxlovid is a co-packaged combination of nirmatrelvir and ritonavir.^517,518^ It is necessary to indicate that both compounds are derivatives of amino acids. Nirmatrelvir is a derivative of proline. It is an orally bioactive inhibitor of SARS-CoV-2 M^pro^ .^519^ Ritonavir is an L-valine derivative that has been applied as an HIV-1 protease inhibitor and as a cytochrome P450 (CYP3A) inhibitor.^520^ Ritonavir does not directly act on SARS-CoV-2 but is used to inhibit CYP3A-mediated metabolism of nirmatrelvir, resulting in increased plasma concentrations of nirmatrelvir.^41^ Owen et al. first developed Nirmatrelvir by optimizing PF-00835231, a potent inhibitor of SARS-CoV M^pro^.^84^ According to their study, niramatrelvir exhibited good selectivity, safety, and protection against infection in a mouse-adapted SARS-CoV-2 model. Moreover, the results from a phase I single ascending dose study in healthy adult participants proved that nirmatrelvir was safe and well tolerated and exhibited a significant boost in plasma concentrations when co-administered with ritonavir.^521^ As M^pro^ is a highly conserved target protein, the antiviral potency of nirmatrelvir does not decrease when treating Alpha (B.1.1.7), Beta (B.1.351), Gamma (P.1), Delta (B.1.617.2), and Omicron (B.1.1.529) SARS-CoV-2 variants.^522^ However, recent preprints have reported that SARS-CoV-2 gains nirmatrelvir resistance after treatment with nirmatrelvir after in vitro culturing.^523,524^ There is also evidence that nirmatrelvir-resistant mutations have been acquired by the SARS-CoV-2 virus circulating in people.^525–527^ Boceprevir belongs to the imino acids and has potential activity against HCV genotype 1. Ma et al. first identified its anti-SARS-CoV-2 activity as an M^pro^ inhibitor and proved its in vitro activity.^96^ This anti-SARS-CoV-2 mechanism and effect was also proved by Fu et al. and Qiao et al..^79,95^ Based on the structure of boceprevir and another peptidomimetic compound, the HCV inhibitor telaprevir, Qiao et al. developed 32 new bicycloproline-containing M^pro^ inhibitors. As a result, two compounds (MI-09 and MI-30) showed excellent antiviral activity in cell-based assays, and significantly reduced lung viral loads and lung lesions in a transgenic mouse model of SARS-CoV-2 infection.^79^ Kneller et al. developed three hybrid peptidomimetic inhibitors, BBH-1, BBH-2, and NBH-2, by splicing components of boceprevir and narlaprevir, and proved their antiviral properties in vitro relative to nirmatrelvir.^86^ A study conducted by Xia et al. also showed the in vitro broad-spectrum coronavirus antiviral effect of two rationally designed inhibitors based on the peptidomimetic compounds GC-376, telaprevir, and boceprevir.^528^ To date, the development of peptidomimetics is the most used strategy in the search of anti-COVID-19 drugs.^529^ Other peptidomimetics, such as talaprevir, MG-132, and MDL-28170, were also found to have M^pro^ binding affinity. The cyclopeptide RTD-1, which has anti-SARS-CoV effects, was found to be safe to support its investigation for treatment of COVID-19.^530^

The effects of other amides, such as lopinavir, on SARS-CoV-2 were also studied. Lopinavir is a dicarboxylic acid diamide which is often used with ritonavir against HIV infections.^531^ Based on the structure of SARS-CoV-2 M^pro^, Zhang et al. first reported it as a candidate drug against COVID-19.^72^ According to a study by Choy et al., lopinavir has in vitro activity against SARS-CoV-2 with an EC_50_ of 26.63 μM.^532^ Niclosamide is a secondary carboxamide resulting from the formal condensation of the carboxy group of 5-chlorosalicylic acid with the amino group of 2-chloro-4-nitroaniline.^533^ It has broad-spectrum antiviral activity, especially against the hepatitis virus, influenza virus, and rotavirus.^534^ It inhibits SARS-CoV-2 virus entry through TMEM16F inhibition and replication through autophagy induction.^535,536^ Weiss et al. showed that niclosamide potency is conserved against the Alpha, Beta, and Delta SARS-CoV-2 variants in Vero TMPRSS2 cells and the strong antiviral activity of niclosamide was validated in a human airway epithelial model.^537^ An inhaled niclosamide formulation was developed and tested in a murine infection model of SARS-CoV-2. Intranasal administration of niclosamide (0.24 mg·kg^−1^·day^−1^) to SARS-CoV-2-infected mice for 10 days improved survival and significantly reduced viral loads.^538^ Darunavir, with a similar anti-HIV effect, belongs to the carbamates.^539^ Computational evidence showed it may interact with the M^pro^ pocket.^540^ Besides, dalcetrapib, an anilide, is a cholesteryl ester transfer protein (CETP) inhibitor that can produce an increase in serum HDL-cholesterol levels and a decrease in serum LDL-cholesterol levels.^541^ Mancek-Keber et al. reported it can disrupt fusion within the RBD and the SARS-CoV-2 S protein.^542^ Niesor et al. claimed it can inhibit M^pro^ activity and viral replication in Vero E6 cells with IC_50_ values of 14.4 ± 3.3 μM and an EC_50_ value of 17.5 ± 3.5 μM.^543^

### Alkaloids

Alkaloids are a complex class of compounds derived from plants with a basic character and bear at least one nitrogen atom, preferably in a heterocycle. Based on their core chemical structures, alkaloids can be classified into various subclasses, such as pyrrolidines, tropanes, quinolines, isoquinolines, and indoles.^544^ Many alkaloids possess biological activity, and have been applied in medicines. Since the COVID-19 outbreak, some alkaloids have been applied in clinical studies to verify their immune regulatory or antiviral effects. Moreover, many alkaloids with potential affinities to SARS-CoV-2 have been screened out by computational methods, and their therapeutic value against COVID-19 has been demonstrated.^545,546^

Quinoline alkaloids bear a common core structure of benzo-pyridine. Their antimalaria and immunomodulatory effects have been broadly investigated.^547,548^ Chloroquine is an aminoquinoline that is substituted at position 7 by chlorine. Since the 1940s, chloroquine has been investigated for the treatment of malaria.^549^ Chloroquine is also used off-label for the treatment of rheumatic diseases, as well as for the treatment and prophylaxis of Zika virus, HIV, dengue fever virus, and coronaviruses SARS-CoV and MERS-CoV.^550,551^ Previous studies revealed it has broad-spectrum antiviral activity by increasing the endosomal pH required for virus/cell fusion, as well as interfering with the glycosylation of cellular receptors of SARS-CoV.^552^ In the early stages of the COVID-19 pandemic, Wang et al. revealed that chloroquine functions both at the entry and at the post-entry stage of SARS-CoV-2 infection, and chloroquine exerts equal inhibitory effects on SARS-CoV-2 in Vero E6 cells infected by SARS-CoV-2 with an EC_50_ of 1.13 μM and an EC_90_ of 6.90 μM.^553^ Since chloroquine is a cheap and safe drug which has been approved for more than 70 years, it is a clinically applicable agent during the COVID-19 pandemic. A multicenter clinical trial conducted by Gao et al. showed that in China, it was effective and had an acceptable safety profile for COVID-19-related pneumonia.^554^ Hydroxychloroquine is similar to chloroquine, but its N-ethyl group at position 2 is hydroxylated. As a less toxic derivative of chloroquine, Liu et al. found that hydroxychloroquine is effective in inhibiting SARS-CoV-2 infection in vitro.^555^ In a pilot observational study, Gautret et al. provided evidence of a beneficial effect of co-administration of hydroxychloroquine with azithromycin in the treatment of COVID-19 and its potential effectiveness in the early reduction of contagiousness.^556^ On 28 March, 2020, the FDA authorized the emergency use of chloroquine and hydroxychloroquine to treat patients with COVID-19. However, further clinical studies provided no conclusive evidence supporting the use of chloroquine or hydroxychloroquine in the treatment of COVID-19. Thus, the FDA emergency use authorization (EUA) for hydroxychloroquine and chloroquine in the treatment of COVID-19 was revoked on 15 June, 2020. Based on the core structure of quinoline, quinazolines are synthetic molecules containing a benzene ring system fused to pyrimidine at two adjacent carbon atoms.^557–559^ Lapatinib, a member of the quinazoline class, has a role as an antineoplastic agent and as a TK inhibitor.^560^ Raymonda et al. showed that lapatinib has the potential to block SARS-CoV-2 infection by a high-throughput screening procedure.^561^ According to their in vitro results, lapatinib could inhibit SARS-CoV-2 RNA replication in pulmonary fibroblasts by over 50,000-fold. Apabetalone is another member of the quinazoline class with benefits in treating COVID-19. Gilham et al. demonstrated it could downregulate the cell surface receptors ACE2 and DPP-4, which are involved in SARS-CoV-2 entry.^562^ Moreover, their results revealed that the inhibitory effects of apabetalone on SARS-CoV-2 infection in vitro are comparable to those of antiviral agents.

Isoquinoline is a benzopyridine in which the nitrogen atom is not directly attached to the benzene ring. The isoquinoline structure occurs in a considerable number of alkaloids in widely separated plant families.^563^ Emetine is a pyridoisoquinoline comprising emetam with methoxy substituents at the 6′-, 7′-, 10-, and 11-positions.^564^ In a previous study, emetine was found to inhibit replication of buffalopox virus (BPXV), bovine herpesvirus 1 (BHV-1) and Newcastle disease virus (NDV).^565^ According to Wang et al., emetine has antiviral effects with an EC_50_ of 0.007 μM, suggesting it is >30-fold more effective than remdesivir (EC_50_: 0.24 μM) against SARS-CoV-2.^566^ Moreover, in vivo pharmacokinetics experiments revealed that emetine was enriched in the lung tissues to effective concentrations at 12 h posttreatment. Interestingly, molecule docking studies suggest that emetine has significant binding affinity toward RdRp (−9.5 kcal/mol), PL^pro^ (−9.0 kcal/mol), the S protein RBD (−8.8 kcal/mol), and M^pro^ (−8.5 kcal/mol) of SARS-CoV-2.^564^ As a multitarget inhibitor of SARS-CoV-2, emetine was recognized to be a more potent drug.^567^ However, there are concerns that need further investigation since cardiovascular complications due to emetine have been reported.^568,569^ Hence, emetine can be used as a lead compound to design high-safety antiviral drugs in the future.^570^ Cepharanthine is a bisbenzylisoquinoline alkaloid from tubers of *Stephania*, which is used as an alopecia drug in Japan.^571^ Its antiviral ability has been verified in vitro against HIV, human T-lymphotropic virus type 1 (HTLV-1), HBV, SARS-CoV, and HCoV-OC43.^572^ The anti-SARS-CoV-2 effect of cepharanthine was verified by Ohashi et al. in vitro.^573^ According to their results, treatment with cepharanthine efficiently decreased the viral RNA concentration in infected cells, and the combination of cepharanthine with nelfinavir exhibited a synergistic effect.

The indole alkaloids with the 2,3-benzopyrrole core structure are important elements of many natural or synthetic molecules with significant biological activity. Melatonin is a therapeutic chemically synthesized form of the pineal indole melatonin with antioxidant properties. It is an effective anti-inflammatory agent and may inhibit SARS-CoV-2-induced cell damage by regulating mitochondrial physiology and enhancing the immune system.^574–576^ A study conducted by Zhai et al. revealed that melatonin could inhibit animal coronavirus infection in cells by reducing viral entry and replication.^577^ Cecon et al. demonstrated that administration of melatonin effectively attenuated severe symptoms and improved survival of human ACE2-expressing mice infected with SARS-CoV-2 by limiting the production of type I and type III interferons in the lungs.^578^ In addition, they demonstrated that melatonin could bind to an allosteric binding site of human ACE2, thus interfering with SARS-CoV-2 entry in endothelial cells.^579^ Indomethacin is a synthetic non-steroidal indole derivative with anti-inflammatory activity and chemopreventive properties.^580^ Amici et al. reported direct antiviral activity of indomethacin by inhibition of viral RNA synthesis against SARS-CoV and canine CoV, without being dependent on the COX inhibitory effect of indomethacin.^581^ Kiani et al. found an increase in percentage inhibition of SARS-CoV-2 to 93% in vitro when co-administered with 100 μM indomethacin compared with administration of ketotifen alone.^582^ An open-label randomized clinical trial of indomethacin for mild and moderate hospitalized COVID-19 patients indicated indomethacin use alongside standard treatment was associated with significant symptomatic relief and improved oxygen saturation levels.^583^ Lufotrelvir is an indolecarboxamide and its metabolic form PF-00835231 has strong and broad-spectrum inhibitory activity against numerous coronavirus 3CL proteases. Boras et al. provided ADME, safety, and in vitro and in vivo antiviral activity data that support lufotrelvir as a potential agent for COVID-19 treatment.^584^ The emvododstat bear core structure of tetrahydropyrido[3,4-b] indole is an orally available potent inhibitor of dihydroorotate dehydrogenase. Luban et al. found that treatment with emvododstat led to a dose-dependent reduction in the levels of SARS-CoV-2 nucleocapsid protein with an EC_50_ of 1.96 nM in infected Vero E6 cells.^585^ Lycorine is an indolizidine alkaloid found in *Sternbergia clusiana* and *Pancratium trianthum*, with inhibitory effects on RdRp activity of coronaviruses.^586^ The antiviral effect of lycorine was verified in Vero E6 cells infected with SARS-CoV-2, with an EC_50_ of 0.31 μM.^587^

### Other small molecules

The anti-COVID-19 effects of various other naturally occurring molecules have also been investigated. Curcumin, a beta-diketone, is a natural dyestuff found in the root of *Curcuma longa*.^588^ As a broad-spectrum antiviral drug, curcumin can not only treat HIV virus, liver poison, and influenza A virus but has also been recognized as a therapeutic agent for COVID-19 as it affects cellular posttranscriptional and posttranslational modifications, thereby limiting viral multiplication.^589,590^ Bormann et al. demonstrated that curcumin effectively neutralizes SARS-CoV-2 at subtoxic concentrations in Vero E6 and human Calu-3 cells.^591^ Treatment significantly reduced SARS-CoV-2 RNA levels in cell culture supernatants. A clinical trial suggested that the use of nanomicelles containing curcumin in COVID-19 patients can accelerate recovery of the acute inflammatory phase, thus controlling the inflammatory response elicited by viral infection.^592^ Further, according to results from a randomized double-blind placebo-controlled trial, nanocurcumin can be effective in increasing oxygen saturation and reducing the severity of symptoms in COVID-19 patients; thus, it can be used as a complementary agent to accelerate the recovery of patients.^593^ Tamoxifen and clomiphene are derived from the natural product stilbene. They belong to the class of stilbenoids and are non-steroidal antiestrogens.^594,595^ Zu et al. showed that tamoxifen and clomiphene strongly antagonized SARS-CoV-2 infection, both in vitro and in vivo.^596^ They functioned by suppressing viral entry in the postbinding stage. In vivo experiments in a mouse model verified that tamoxifen and clomiphene effectively suppress infection of not only wild-type but also mutant SARS-CoV-2 variants such as P.1.351 and P.1.617.^594^ Ivermectin is a natural and broad-spectrum anti-infective agent found in *Streptomyces avermitilis*, and it can inhibit the replication of HIV-1, Newcastle disease virus and dengue virus.^597^ Research indicated it exerts inhibitory effects on SARS-CoV-2 replication in the early stages of infection. Ivermectin has recently been reported as a potent inhibitor of SARS-CoV-2 infection, with an excellent ability to reduce viral RNA levels in Vero-hSLAM cells.^598^ Carrageenan is a polysaccharide found in red algae with antiviral effects. Carrageenans, which are used in broadly used nasal and mouth sprays, have the potential to serve as first-line therapeutics to inhibit infection and transmission of SARS-CoV-2.^599^ Schutz et al. identified the mechanisms underlying the antiviral activity of one nasal and one mouth spray through in vitro assays.^600^ This antiviral effect was also observed by Froba et al. against several SARS-CoV-2 variants (Alpha, Beta, Gamma, and Delta).^599^ An aurothioglucose named auranofin exerted inhibitory effects on SARS-CoV-2 in Huh7 human liver cells for more than 24 h.^601^ It also suppressed the papain-like proteinase activity of SARS-CoV-2 in vitro with an IC_50_ of 0.75 ± 0.13 µM, and reduced the binding of the S protein of SARS-CoV-2 and human ACE2 in vitro with an IC_50_ of 22.2 ± 2.8 µM.^602^ Hypericin is an anthraquinone derivative that is naturally found in the yellow flower of *Hypericum perforatum*. It was identified as a candidate drug for COVID-19 therapy due to its inhibitory effects on SARS-CoV-2 PL^pro^ in vitro.^603,604^

The effects of other synthetic small molecules on COVID-19 were also studied. For example, camostat, a benzoate ester, is a synthetic serine protease inhibitor.^605^ Hoffmann et al. demonstrated that camostat treatment significantly reduced Calu-3 infection with wild-type SARS-CoV-2 by blocking TMPRSS2 of target cells. According to their results, camostat reduced SARS-CoV-2 entry into cells with an EC_50_ of 1 µM and EC_90_ of 5 µM.^606^ In a retrospective analysis of 371 adult patients with COVID-19 pneumonia, Sakr et al. concluded that camostat treatment could be beneficial to critically ill COVID-19 patients.^607^ However, Chupp et al. claimed that camostat was not associated with a reduction in nasopharyngeal SARS-COV-2 viral load compared to placebo.^608^ Nafamostat, an analog of camostat and a member of the guanidines, also has potential anti-COVID-19 effects by blocking TMPRSS2 on target cells.^609^ Li et al. reported that nafamostat reduced SARS-CoV-2 infection in primary human airway epithelial cells and in the Calu-3 2B4 cell line, and exhibited greater antiviral efficiency compared with camostat.^610^ Moreover, they demonstrated that intranasal nafamostat treatment prior to or shortly after SARS-CoV-2 infection significantly reduced weight loss and lung tissue titers of mice infected by SARS-CoV-2. Jang et al. reported three cases of COVID-19 pneumonia who progressed while using antiviral drugs, needed supplementary oxygen therapy, and improved after treatment with nafamostat. However, according to the results of a phase Ib/IIa clinical study, no evidence of anti-inflammatory, anticoagulant, or antiviral activity of intravenous nafamostat in hospitalized COVID-19 patients was provided.^611^ The negative outcomes of the abovementioned TMPRSS2 inhibitors raise questions about the effectiveness of this target. It is worth noting that blocking TMPRSS2 might not function well when the virus has already infected the human body and caused symptoms. Thus, it is recommended to use TMPRSS2 inhibitors in the early stage of COVID-19 or to use them in combination with other anti-viral drugs. Amantadine is a synthetic amine with antiviral effects by interfering with the function of the transmembrane domain of the viral proteins.^612^ Its antiviral ability against SARS-CoV-2 has been tested in vitro in a study conducted by Fink et al., and was found to have an IC_50_ of around 100 μM.^613^ According to a case report, the use of amantadine may reduce the toxic effects of COVID-19, including ARDS, viral replication, and ventilator dependency.^614,615^ Currently, two phase III clinical trials determining if amantadine brings benefits in patients with COVID-19 are ongoing (NCT04952519; NCT04894617). Brilacidin, a non-peptidic small molecule mimetic of defensin, which is a type of host defense protein/peptide with antibacterial and antiviral activities, is also referred to as a SARS-CoV-2 inhibitor.^616,617^ Bakovic et al. demonstrated that brolacidin could impact viral entry and disrupt viral integrity, thus exerting inhibitory effects on SARS-CoV-2 infection in Calu-3 and Vero E6 cells.^618^ Other synthetic molecules, such as GLPG-0187 (a sulfonamide) and the cyclohexanone SIMR-2418, are also potential inhibitors of SARS-CoV-2 with proved in vitro antiviral effects.^619,620^

## Small molecule drugs in clinical development

### Approved/authorized products

So far, the FDA has approved two small molecular drugs, remdesivir (Veklury) and baricitinib (Olumiant), for the treatment of COVID-19.^31,621^ Remdesivir, which was developed by Gilead, is approved for the treatment of mild to moderate COVID-19 in adults and pediatric patients.^32^ Baricitinib, which was developed by Eli Lilly, is approved for the treatment of COVID-19 in hospitalized adults requiring supplemental oxygen, non-invasive or invasive mechanical ventilation, or extracorporeal membrane oxygenation (ECMO).^319^ In addition, the FDA has granted EUA for the use of several unapproved drugs against COVID-19, which include two oral antiviral pills, nirmatrelvir/ritonavir (Paxlovid) and molnupiravir (Lagevrio).^622,623^ Four drugs, favipiravir (AVIFAVIR), proxalutamide, azvudine, and VV116 have been approved in Russia, Paraguay, China, and Uzbekistan, respectively (Table 1).^45,288^

**Table 1:** Approved/authorized products

Agent	Trade name	Company	Mechanism	Structural category	Indication	Suitable crowd	Recommended dosage	Side effects	First approved date, approved region
Remdesivir	Veklury	Gilead Sciences	RdRp inhibitor	Nucleoside/Nucleotide Analogs	Mild-to-moderate and severe COVID-19	Adults and pediatric patients (older than 12 and weighing at least 40 kg)	100–200 mg/day	Severe headache, pounding in your neck or ears, etc	2020/10 USA, Japan, EU
Baricitinib	Olumiant	Eli Lilly Company	JAK2 inhibitor, JAK1 inhibitor	Nucleoside/Nucleotide Analogs	Severe COVID-19	Hospitalized adults	2–4 mg/day	Serious venous thrombosis	2022/5 USA, Japan
Nirmatrelvir/ Ritonavir	Paxlovid	Pfizer	CYP3A inhibitor, M^pro^ inhibitor	Amides	Mild-to-moderate COVID-19	Adults and pediatric patients	300 mg nirmatrelvir with 100 mg ritonavir twice daily	Altered or impaired sense of taste, diarrhea, etc	2022/4 USA, China, Japan, UK, EU, Israel, Korea
Molnupiravir	Lagevrio	Merck Sharp & Dohme Co	RdRp inhibitor	Nucleoside/Nucleotide Analogs	Mild-to-moderate and severe COVID-19	Adult patients	800 mg twice daily	Diarrhea, dizziness, and nausea	2021/11 UK, USA, Japan, Singapore, India
Favipiravir	Avifavir,	Zhejiang Hisun Pharmaceutical Co	RdRp inhibitor	Nucleoside/Nucleotide Analogs	Mild to moderate COVID-19	Hospitalized patients	1.6–1.8 g/day	Diarrhea, decreased white blood cells count, etc	2020/5 Russia
Proxalutamide	Proxalutamide	Corpometria Institute	AR antagonist	Azoles	Mild-to-moderate COVID-19	Hospitalized patients	200 mg/ day	Fatigue, nausea, dizziness, loss of appetite, etc	2021/7 Paraguay
VV116	Mindvy	Shanghai JunTop Biosciences Co	RdRp inhibitor	Nucleoside/Nucleotide Analogs	Mild-to-moderate COVID-19	N/A^*^	N/A^*^	Back pain, chest tightness, chills, cough, etc	2022/5 Uzbekistan
Azvudine	Azvudine	Genuine Biotech Co., Ltd	RdRp inhibitor	Nucleoside/Nucleotide Analogs	Mild-to-moderate COVID-19	Adult patients	5 mg/day	Dizziness, nausea, etc	2022/7 China

*No sufficient information available.

*Remdesivir* was approved by the FDA on May 1, 2020 as the first treatment for COVID-19. On May 7, 2020, it was approved for emergency situations by the Pharmaceuticals and Medical Devices Agency (PMDA) of Japan, and its use was authorized by the EMA on July 3, 2020. This approval is supported by the data from three randomized, controlled clinical trials that included patients hospitalized with mild to severe COVID-19. In detail, the first adaptive, randomized, double-blind, placebo-controlled trial to evaluate the safety and efficacy of remdesivir in hospitalized adults diagnosed with COVID-19 (ACTT-1) was supported by the National Institute of Allergy and Infectious Diseases (NIAID) (NCT04280705). The results of this trial were published by Beigel et al..^277^ In brief, 1062 patients (541 assigned to the remdesivir group and 521 to the placebo group) were included in this trial. Participants who received remdesivir had a shorter recovery time (10 days) compared with the placebo group (15 days). The second study to evaluate the antiviral activity of remdesivir in participants with moderate COVID-19 compared with standard care treatment was supported by Gilead Sciences (NCT04292730). According to the results presented by Spinner et al., patients who received 5-day remdesivir treatment had a significantly better clinical status than those who received standard care at 11 days after initiation of treatment.^624^ The third study to evaluate the safety and antiviral activity of remdesivir treatment in patients with severe COVID-19 was supported by Gilead Sciences (NCT04292899). According to the results, improvements in symptoms were similar in both groups of patients treated with 5-day remdesivir and 10-day remdesivir.^625^

Besides the above trials, a double-blind, randomized, placebo-controlled phase III trial conducted at 63 hospitals across five countries (Japan, Mexico, Singapore, South Korea, and the USA) by the NIAID (NCT04492475; EudraCT2020-003510-12) also revealed the anti-COVID-19 effects of remdesivir. Participants involving symptomatic, non-hospitalized patients with COVID-19 who are at high risk for disease progression (age ≥ 60 years, obesity, or certain co-existing medical conditions) were randomly assigned to the remdesivir group or the placebo group.^626^ A 3-day course of remdesivir had an acceptable safety profile. Compared with the placebo group, the remdesivir group had an 87% lower risk of COVID-19-related hospitalization or death and an 81% lower risk of COVID-19-related medically attended visits or death. However, according to another phase III, randomized, controlled, open-label trial (DisCoVeRy) conducted at 48 sites in Europe (France, Belgium, Austria, Portugal, and Luxembourg) (NCT04315948; EudraCT2020-000936-23), no clinical benefit from remdesivir treatment was observed in hospitalized COVID-19 patients with symptoms for more than 7 days who required oxygen support.^627,628^ The authors speculated that the discrepancy between their results and those from ATCC-1 might be explained by the differences in study populations.^277^ Among the patients without requirement of oxygen support in the DisCoVeRy trial, remdesivir significantly delayed the need for new mechanical ventilation or ECMO or death, consistent with what was reported in ACTT-1. In addition, a randomized, double-blind, placebo-controlled, multicenter trial of remdesivir carried out at ten hospitals in China (NCT04257656) also indicated that remdesivir was not associated with statistically significant clinical benefits in adult patients admitted to the hospital for severe COVID-19.^629^

*Barcitinib* was approved by the FDA on May 10, 2022 for treatment of COVID-19 in hospitalized adults requiring supplemental oxygen, non-invasive or invasive mechanical ventilation, or ECMO, and authorized under EUA for the same indication for pediatric patients (2–17 years old) in the USA.^630^ Barcitinib has also been approved by the PMDA of Japan on April 23, 2021 for treatment of pneumonia caused by COVID-19 (limited to patients requiring supplemental oxygen), and a marketing authorization application for barcitinib has been submitted in the European Union. The approval of barcitinib in both countries was supported by data from two clinical trials conducted by the NIAID.^41^ ACTT-2 (NCT04401579) is a randomized, double-blind, placebo-controlled trial evaluating if combination with baricitinib could improve the effects of remdesivir against COVID-19 in hospitalized adults. According to the results, combination treatment with the anti-inflammatory drug baricitinib and the antiviral drug remdesivir was safe and superior to remdesivir alone for the treatment of hospitalized patients with COVID-19 pneumonia.^319^ The clinical trial COV-BARRIER is a randomized, double-blind, placebo-controlled, parallel-group phase III study to verify if baricitinib is effective in hospitalized patients with COVID-19 (NCT04421027). First, the efficacy and safety results of baricitinib plus standard care (include systemic corticosteroids and remdesivir) in hospitalized adults with COVID-19 from 101 centers across 12 countries were presented.^41^ Baricitinib plus standard care lowered the absolute all-cause mortality risk to 5% at 28 days and 4.9% at 60 days. Another study reported the results from a critically ill cohort in COV-BARRIER not included in the main phase III trial.^631^ This study was conducted across 18 hospitals in Argentina, Brazil, Mexico, and the USA. According to the results, in critically ill hospitalized COVID-19 patients who received invasive mechanical ventilation or ECMO, combination treatment with baricitinib and standard care lowered the absolute all-cause mortality risk to 5% at 28 days and 17% at 60 days. This result is in line with the previously reported results of baricitinib in patients with less severe COVID-19.

*Molnupiravir*, which was developed by Merck, was first approved by the MHRA of the UK on November 4, 2021 for treatment of mild to moderate COVID-19 in adults with a positive SARS-COV-2 diagnostic test and who have at least one risk factor for developing severe illness.^632^ Further, it was authorized for the same indications by FDA on December 23, 2021. On the next day, it was approved for emergency use by the PMDA of Japan for treatment of diseases caused by SARS-CoV-2 infection.^38,633^ The approval and EUA of molnupiravir were mainly based on data from two clinical trials. The first one is a phase IIa double-blind, placebo-controlled, randomized trial evaluating the safety, tolerability, and antiviral efficacy of molnupiravir in patients with COVID-19 (NCT04405570). At the end of the 4-week study, the proportion of participants who achieved viral RNA clearance was higher in the 800-mg molnupiravir group (92.5%) than in the placebo group (80.3%).^634^ Moreover, the proportion of nasopharyngeal swabs containing infectious virus and the time to eliminate SARS-CoV-2 RNA were decreased. These results provide strong biological evidence indicating that molnupiravir can be used as an oral agent for COVID-19 treatment during the early stages of the disease. Another one is a phase II/III double-blind, placebo-controlled, randomized, multicenter clinical trial, MOVe-OUT, evaluating the safety, tolerability, and antiviral efficacy of molnupiravir in non-hospitalized adults with COVID-19 (NCT04575597). As shown by Bernal et al., data from the MOVe-OUT phase III trial indicate that initial treatment with molnupiravir within 5 days after the onset of symptoms reduces the risk of hospitalization for any cause or death through day 29.^635^

*Nirmatrelvir/ritonavir*, developed by Pfizer, is a co-packaged combination that is used to treat SARS-CoV-2 infection.^636^ The FDA issued an EUA for nirmatrelvir/ritonavir for the treatment of mild to moderate COVID-19 in adults and pediatric patients (≥12 years of age and weighing ≥40 kg) with SARS-CoV-2 infection and those patients who are at high risk of progression to severe COVID-19 on December 22, 2021. Nirmatrelvir/ritonavir was approved in Israel (December 26, 2021), Korea (December 27, 2021), UK (December 31, 2021), and the EU (January 28, 2022) for the treatment of COVID-19 in adults who do not require supplemental oxygen and are at increased risk of developing severe COVID-19. On February 10, 2022, the PMDA of Japan specially approved the use of Pfizer oral medicine, and signed a purchase agreement with Pfizer for 2 million people. On February 11, 2022, the National Medical Products Administration of the People’s Republic of China approved Paxlovid for emergency use in adults and adolescents with mild, common forms of COVID-19 within 5 days of onset and associated with severe risk factors for progression. The primary data supporting Paxlovid’s EUA came from EPIC-HR (NCT04960202), a randomized, double-blind, placebo-controlled clinical phase II/III trial of nirmatrelvir plus ritonavir in the treatment of non-hospitalized symptomatic adults with laboratory-confirmed SARS-CoV-2 infection. Results from this trial demonstrated the efficacy of oral administration of nirmatrelvir (300 mg) with ritonavir (100 mg) every 12 h for 5 days.^519,637,638^ Among non-hospitalized adults at high risk of progression to severe disease, treatment with nirmatrelvir plus ritonavir resulted in an 89.1% relative risk reduction of COVID-19-related hospitalization or death from any cause compared with the placebo group by day 28.^518,519^ Currently, the EPIC-HR trial is ongoing, and further data like the proportion of all-cause death will be published later. Besides, the phase II/III EPIC-SR trial which compares nirmatrelvir plus ritonavir and placebo for the treatment of non-hospitalized, symptomatic adults with COVID-19 who are at low risk of progressing to severe illness is also ongoing (NCT05011513). The phase II/III EPIC-PEP trial, which evaluates the efficacy and safety of two nirmatrelvir plus ritonavir regimens in preventing symptomatic SARS-COV-2 infection in adult household contacts of people infected with SARS-COV-2, is still recruiting participants (NCT05047601), and the EPIC-Pedstrial, a study of oral nirmatrelvir/ritonavir in non-hospitalized COVID-19 pediatric patients at risk for severe disease, is also recruiting participants. Further clinical trials are being conducted not only by Pfizer, but also in China, Japan, and other countries to further prove the safety and effectiveness of the drug.^639^

*Favipiravir* has been granted a conditional marketing authorization by the Russian Ministry of Health based on the interim results of a phase II/III clinical trial in May 2020^640^ (NCT04434248). In this adaptive, multicenter, open-label, randomized, phase II/III clinical trial, hospitalized patients with moderate COVID-19 were randomized at a 1:1:1 ratio to receive favipiravir with different dosages or standard care. According to their results, viral clearance was achieved in 62.5% of patients in the favipiravir groups and in 30% of patients in the standard care group on day 5, demonstrating a rapid antiviral response against SARS-CoV-2. However, according to the data from a randomized, double-blind, multicenter, and placebo-controlled trial in Saudi Arabia, favipiravir was not associated with faster viral clearance or a better clinical outcome when initiated within 5 days after onset of COVID-19 symptoms in adults with mild COVID-19.^641^ This conclusion was also drawn based on data from a prospective, randomized, open-label, multicenter trial of favipiravir for the treatment of COVID-19 at 25 hospitals across Japan^642^ (jRCTs041190120).

*Proxalutamide*, developed by Kintor Pharmaceuticals, has exhibited efficiency in preventing COVID-19 in a randomized, double-blind, placebo-controlled, multiregional clinical trial of Proxalutamide for hospitalized COVID-19 patients clinical trial conducted in Brazil. This trial was carried out with two different arms, the Northern Brazil arm (NCT04728802) and the Southern Brazil arm (NCT05126628). According to the combined results published by Cadegiani et al., the recovery rate was 121% higher in the proxalutamide group than in the placebo group at day 14 and 81% higher at day 28.^493^ Moreover, the all-cause mortality rate was 80% lower in the proxalutamide group than in the placebo group at day 14 and 78% lower at day 28. However, these results were suspicious since the fatality rate was as high as 49.4% in the placebo group and the trial was conducted very quickly. In reply to these suspicions, Cagegiani claimed the fatality rate was high due to the Gamma variant’s wide spread in Brazil at the time; about 43% of the hospitalized COVID-19 patients in the state of Amazonas were dying, according to official data. Recruitment was rapid because word got out that patients in the proxalutamide trial were recovering within days. Then, a randomized, double-blind, placebo-controlled clinical trial of proxalutamide was conducted in Brasilia, Brazil in men with COVID-19 in an outpatient setting (NCT04446429). Proxalutamide treatment reduced the rate of hospitalization by 91% in this trial.^643^ However, since tests of antiandrogens in COVID-19 patients were not encouraged except in the Brazilian trial,^644^ the anti-COVID-19 function of proxalutamine remains to be verified by clinical trials of other countries and organs (NCT04870606; NCT05009732). Also, its mechanism should be discussed in further studies.

*VV116* was developed by Shanghai JunTop Biosciences Co., Ltd. According to data from an open, prospective cohort study of VV116 in Chinese participants infected with the SARS-CoV-2 Omicron variant (NCT05242042), participants who received VV116 within 5 days after the first positive PCR test of SARS-CoV-2 had a shorter viral shedding time than participants in the control group (8.56 vs. 11.13 days).^289^ VV116 exhibits a wide distribution in target organs of SARS-CoV-2 in rats and dogs.^645^ In this regard, VV116 might compensate for the liver-targeting limitation of remdesivir. VV116 has been approved for the treatment of COVID-19 in Uzbekistan and is being investigated in several phase III clinical trials in patients with COVID-19 (NCT05242042; NCT05279235; NCT05341609).

*Azvudine* was developed by Genuine Biotech Co., Ltd. According to data from a randomized, open-label, controlled clinical trial performed in China (ChiCTR2000029853), azvudine treatment plus standard care shortens the mean time of the first nucleic acid negative conversion in mild and common COVID-19 patients.^282^ Moreover, a randomized, single-arm clinical trial revealed that azvudine treatment cured COVID-19 patients, with the duration of nucleic acid negative conversion of 3.29 ± 2.22 days and hospital discharge at 9.00 ± 4.93 days.^281^ On July 25, 2022, azvudine was conditionally approved for the treatment of COVID-19 in China.

### Candidates under phase III/IV clinical trials

*Nitazoxanide* is a broad-spectrum antiviral agent in vitro, so it was a logical choice to analyze its anti-SARS-CoV-2 effects (Table 2).^534^ According to data from a phase II multicenter, randomized, double-blind, placebo-controlled trial conducted in Brazil (NCT04552483), early nitazoxanide therapy in patients with mild COVID-19 reduced the viral load compared with the placebo group.^488^ A pilot proof-of-concept randomized double-blind clinical trial in patients hospitalized with moderate to severe COVID-19 also concluded that nitazoxanide is superior to placebo (NCT04348409), since patients in the nitazoxanide group had a lower mortality rate and faster hospital discharge compared to the placebo group.^489^ Based on these studies, several phase III trials were conducted in different countries. Among them, a randomized double-blind placebo-controlled clinical trial in 36 centers in the USA has given corresponding results (NCT04486313). According to the findings, nitazoxanide reduced the relative risk of progressing to severe illness in mild or moderate COVID-19 patients,^646^ suggesting it may reduce the progression to severe illness in high-risk participants. However, there is no significant difference in sustained clinical recovery time between the nitazoxanide and placebo treatment groups. The efficiency of nitazoxanide in patients should be tested by larger phase III trials with adequate statistical power (NCT04343248; NCT05157269; NCT05157243).

**Table 2:** Candidates under phase III/IV clinical trials

Agent	Investigator	Mechanism	Structural category	Clinical Trial Registrations	Current stage & region	Indication
Pacritinib (Vonjo)	CTI BioPharma	JAK2 inhibitor	Other molecules	NCT04404361 (Terminated)	Phase III, USA	Severe COVID-19
Danoprevir Sodium (Danoprevir)	Huoshenshan Hospital	HCV protease (NS3/4A) inhibitor	Amides	NCT04345276 (Completed)	Phase IV, China	COVID-19
Fostamatinib disodium (Tavalisse, Tavlesse)	Rigel Pharmaceuticals	Syk inhibitor	Nucleoside/ Nucleotide Analogs	NCT04629703 (Recruiting)	Phase III, USA, Argentina, Brazil, Mexico, Peru	COVID-19 with severe ARDS
Apremilast (Otezla, Aplex)	Amgen, UMC Utrecht	PDE4 inhibitor	Alkaloids	NCT04590586 (Completed) NCT02735707 (Recruiting)	Phase III, USA, Argentina, Brazil, Russian, Germany, Japan, etc.	COVID-19
Enisamium iodide (Amizon)	Joint Stock Company “Farmak”, UMC Utrecht	RdRp inhibitor	Amides	NCT04682873 (Completed)	Phase III, Ukraine, Argentina, Brazil, Canada, Chile, Colombia	COVID-19
Rivaroxaban (Xarelto)	Yaounde Central Hospital	coagulation factor Xa inhibitor	Azoles	NCT04715295 (Recruiting) NCT04394377 (Completed)	Phase IV, Cameroon, Brazil	COVID-19
Ciclesonide (Alvesco)	Covis Pharma S.à.r.l, Cambridge University Hospitals NHS Foundation Trust, ANRS, Emerging Infectious Diseases, University Hospital, Bordeaux	Anti-inflammation	Steroids	NCT04377711 (Completed) NCT04870333 (Recruiting) NCT04920838 (Recruiting) NCT04356495 (Completed)	Phase III, USA, UK, Burkina Faso, Guinea, France	COVID-19
Nitazoxanide (Alinia, Nizonide)	Romark Laboratories L.C, University of Cape Town	PFOR inhibitor	Azoles	NCT04486313 (Completed) NCT04523090 (Recruiting)	Phase III, USA, South Africa	Mild or moderate COVID-19
Camostat Mesilate (Foipan)	Ono Pharmaceutical Co., Ltd, KU Leuven, Daewoong Pharmaceutical Co, LTD, National Institute of Allergy and Infectious Diseases (NIAID)	Protease inhibitor	Other molecules	NCT04657497 (Completed) NCT04730206 (Recruiting) NCT04713176 (Recruiting) NCT04518410 (Active, not recruiting)	Phase III, Japan, Belgium, Korea	COVID-19
ABX-464 (Obefazimod)	Abivax SA	RT inhibitor	Alkaloids	NCT04393038 (Terminated)	Phase III, Belgium, Brazil, Germany, Italy, Mexico, Spain, United Kingdom	COVID-19
Emvododstat	PTC Therapeutics, Inc	VEGFA inhibitor	Alkaloids	NCT04439071 (Recruiting)	Phase III, USA, Belgium, Australia, Brazil, Colombia, France	COVID-19
Tradipitant (Tradipitant)	Vanda Pharmaceuticals, Inc	NK1R inhibitor	Azoles	NCT04326426 (Unknown)	Phase III, USA	COVID-19
Losmapimod	Fulcrum Therapeutics, Inc	p38-γ MAPK inhibitor	Amides	NCT04511819 (Terminated)	Phase III, USA, Brazil, Mexico, Peru	Moderate COVID-19
Zavegepant (Vazegepant)	Biohaven Pharmaceuticals, Inc	CGRP inhibitor	Azoles	NCT04346615 (Recruiting)	Phase III, USA	COVID-19
Sabizabulin	Veru Inc	Tubulin polymerization inhibitor	Azoles	NCT04842747 (Active, not recruiting) NCT04388826 (Completed)	Phase III, USA, Argentina, Brazil, Bulgaria, Colombia, Mexico	COVID-19
Opaganib (Yeliva)	RedHill Biopharma Limited. Shaare Zedek Medical Center	SPHK2 inhibitor	Amides	NCT04467840 (Completed) NCT04435106 (Completed)	Phase III, USA, Brazil, Colombia, Israel, Italy, Russian, UK, ect.	Severe COVID-19
Vidofludimus calcium	Immunic AG	DHODH inhibitor	Amides	NCT04379271 (Completed)	Phase III, Bulgaria, Germany	COVID-19
Tempol	Adamis Pharmaceuticals Corporation	Coagulation factor inhibitor, Inflammatory inhibitor	Other molecules	NCT04729595 (Recruiting)	Phase III, USA	COVID-19
Apabetalone	Resverlogix Corp	BRD4 inhibitor	Alkaloids	NCT04894266 (Recruiting)	Phase III, Canada	COVID-19
AZD7986 (Brensocatib)	University of Dundee	CTSC inhibitor	Amides	NCT04817332 (Completed)	Phase III, United Kingdom	COVID-19
Carrimycin (Bite)	Shenyang Tonglian Group CO., Ltd	50S ribosomal subunit inhibitor	Other molecules	NCT04672564 (Recruiting)	Phase III, USA, Argentina, Brazil, Colombia, India, ect.	Severe COVID-19
Indomethacin (Indocid, Indocin)	Sen-Jam Pharmaceutical	Phospholipase A2 inhibitor	Alkaloids	NCT05007522 (Recruiting)	Phase III, Nepal	COVID-19 Respiratory
Brexanolone (Zulresso)	Sage Therapeutics	GABAAR modulator	Steroids	NCT04537806 (Terminated)	Phase III, USA	ARDS due to COVID-19
Silymarin	F.D. Roosevelt Teaching Hospital with Policlinic Banska Bystrica	TMPRSS2 inhibitor	Flavonoids	NCT04816682 (Recruiting)	Phase IV, Slovakia	COVID-19
Sofosbuvir (Sovaldi)	Alexandria University	RdRp inhibitor	Nucleoside/ Nucleotide Analogs	NCT04773756 (Completed)	Phase IV, Egypt.	COVID-19
Quercetin	Ministry of Health, Saudi Arabia	Mpro, PLpro, and 6LU7 proteinase inhibitor	Flavonoids	NCT04468139 (Recruiting)	Phase IV, Pakistan, Saudi Arabia, Indonesia	COVID-19
Luteolin	University Of Perugia	Anti-inflammation, Antioxidant	Flavonoids	NCT04853836 (Completed)	Phase IV, Italy	COVID-19
EGCG (Previfenon)	MELISA Institute Genomics & Proteomics Research SpA	ACE2 receptor inhibitor, Mpro inhibitor	Flavonoids	NCT04446065 (Not yet recruiting)	Phase III, USA	COVID-19
Andrographolide	Mahidol University, Swedish Herbal Institute AB	PLC gamma2/ PKC inhibitor, PI3K/AKT-MAPK inhibitor	Terpenoids	NCT05019326 (Recruiting) NCT04847518 (Recruiting)	Phase III, Thailand, USA	mild and asymptomatic COVID-19
Cannabidiol (Epidiolex, Epidyolex)	University of Sao Paulo, Cardiol Therapeutics Inc	Mpro inhibitor, TMPRSS2 inhibitor CB2R inhibitor	Terpenoids	NCT04504877 (Completed) NCT04615949 (Recruiting)	Phase III, Brazil, USA, Germany, Mexico	COVID-19
Methylprednisolone (Medrol, Depo-Medrol, Solu-Medrol)	Cairo University	Anti-inflammation	Steroids	NCT05062681 (Recruiting)	Phase IV, Pakistan, Italy, Bangladesh, Egypt	COVID-19
Curcumin (Curcuplex-95)	XYMOGEN	COX inhibitor	Other molecules	NCT04802382 (Active, not recruiting)	Phase III, Israel	COVID-19
Cholecalciferol	Fundación para la Investigación Biosanitaria del Principado de Asturias	ACE2 receptor inhibitor	Steroids	NCT04552951 (Recruiting)	Phase IV, Spain	COVID-19
Calcifediol	Fundación para la Investigación Biosanitaria del Principado de Asturias	VDR activator	Steroids	NCT04552951 (Recruiting)	Phase IV, Spain	COVID-19
Chloroquine (Aralen)	Tanta University, Centro de Estudos e Pesquisa em Emergencias Medicas e Terapia Intensiva, Medical University of Vienna	ACE2 inhibitor Phospholipase A2 inhibitor, TLR inhibitor	Alkaloids	NCT04353336 (Completed) NCT04420247 (Completed) NCT04447534 (Completed) NCT04351724 (Recruiting) NCT04351295 (Completed) ect.	Phase III, Egypt, Brazil, Austria	COVID-19
Hydroxychloroquine (Plaquenil)	Hospital Alemão Oswaldo Cruz, St. Francis Hospital	ACE2 inhibitor Phospholipase A2 inhibitor, TLR inhibitor	Alkaloids	NCT04466540 (Completed) NCT04370782 (Completed)	Phase IV, Brazil, Spain, Turkey, USA, Mexico	COVID-19
Amantadine (Gocovri, Symadine, Symmetrel)	Noblewell, Copenhagen University Hospital, Hvidovre. Independent Public Clinical Hospital No. 4 in Lublin	Ion-channel inhibitor	Other molecules	NCT04952519 (Recruiting) NCT04894617 (Recruiting) NCT04854759 (Recruiting)	Phase III, Poland, Denmark	Moderate or Severe COVID-19

*Camostat mesylate*, an oral TMPRSS2 inhibitor, is used to treat chronic pancreatitis and reflux esophagitis. In a phase I clinical study (NCT04451083), it was shown to be safe and tolerable at a high dosage in healthy male subjects.^647^ A preprint article reported results of a phase II randomized, double-blind, placebo-controlled trial of camostat mesylate involving 70 COVID-19 outpatients (NCT04353284). In this trial, more rapid resolution of COVID-19 symptoms and amelioration of the loss of taste and smell was observed in the camostat group compared to the placebo group.^608^ However, treatment with camostat did not appear to be associated with a reduced nasopharyngeal SARS-COV-2 viral load in this trial. However, since camostat functions by inhibiting viral entry, it would possibly lead to similar viral load in the upper respiratory tract of patients in both groups. Thus, additional clinical trials are needed with a larger sample size to obtain more information about other symptomatic outcomes of camostat in early COVID-19. Currently, eight phase III clinical trials are registered at the ClinicalTrials.gov website, but none of their results have been reported.

*Ciclesonide*, a glucocorticoid, is applied for the treatment of obstructive airway diseases including asthma and chronic obstructive pulmonary disease.^648^ Based on its anti-inflammatory effect, it was hypothesized that it could decrease the symptom burden of COVID-19 in patients with prominent respiratory symptoms.^649^ In the phase II/III randomized, double-blind, placebo-controlled trial CONTAIN, it was analyzed if ciclesonide accelerates recovery from COVID-19 in outpatients (NCT04435795). However, the combination of inhaled and intranasal ciclesonide was not associated with an appreciable increase in symptom resolution among healthy young adults with COVID-19 who presented with cough, dyspnea, or fever compared with the placebo group according to the data of the phase II trial.^650^ The further phase III clinical trial was terminated since the researchers could not meet enrollment targets in Canada. Another phase III study evaluating the efficacy of inhaled ciclesonide was conducted in non-hospitalized participants with symptomatic COVID-19 (NCT04377711). In brief, 400 participants were enrolled and randomized in the ciclesonide arm or the placebo arm. The median time to alleviation of all COVID-19-related symptoms was 19.0 days in the ciclesonide arm and 19.0 days in the placebo arm, which suggested ciclesonide did not reduce the time to alleviate COVID-19-related symptoms.^651^ Furthermore, a meta-analysis evaluating the effect of inhaled ciclesonide in COVID-19 outpatients was conducted by Hsu et al..^652^ By searching and analyzing data from four randomized controlled trials, the authors concluded that inhaled ciclesonide could not relieve the symptoms for COVID-19 outpatients.

*Rivaroxaban* is a direct inhibitor of the coagulation factor Xa with anticoagulant activity.^653^ Since COVID-19 is associated with both venous and arterial thrombotic complications, prophylactic anticoagulation is widely recommended for hospitalized patients with COVID-19.^654^ Thus, several phase III/IV clinical trials have evaluated its anticoagulant ability in COVID-19 patients. Among them, ACTION is an academic-led, pragmatic, multicenter, open-label, randomized phase IV clinical trial conducted in Brazil (NCT04394377). It was designed to determine whether therapeutic anticoagulation with rivaroxaban improves clinical outcomes in hospitalized patients with COVID-19 and elevated D-dimer levels compared with standard prophylactic anticoagulation.^655^ As a result, in-hospital therapeutic anticoagulation with rivaroxaban or enoxaparin followed by rivaroxaban to day 30 did not improve clinical outcomes and increased bleeding compared with prophylactic anticoagulation,^656^ suggesting that a dosage of 20 mg rivaroxaban per day should be avoided as a routine anticoagulation strategy in hospitalized COVID-19 patients (NCT04662684). However, another phase III open-label, multicenter, randomized trial conducted at 14 centers in Brazil evaluated post-discharge thromboprophylaxis effects of rivaroxaban versus no anticoagulation in COVID-19 patients (NCT04662684).^654^ It was found that thromboprophylaxis with 10 mg/day rivaroxaban for 35 days improved clinical outcomes compared with no extended thromboprophylaxis in post-discharge patients with a high risk for venous thromboembolism. This study revealed that low-dose rivaroxaban at the time of hospital discharge and for another 35 days in the right patient population improves clinical outcomes. Despite these results, future trials with multiple study populations (such as COVID-19 patients at high risk of disease progression, or mild COVID-19 patients) are warranted to confirm the above findings, and the function of rivaroxaban when combined with different antiviral candidates. Thus, several related phase III/IV clinical studies are in active or recruiting status at present (NCT04351724; NCT04324463; NCT04715295).

*Ivermectin* is an anti-infective agent with activity against several parasitic nematodes and scabies and is the treatment of choice for onchocerciasis (river blindness). Since its anti-SARS-CoV-2 ability was observed in vitro and in animal models,^598,657^ ivermectin has attracted much attention in the fight against COVID-19. It has been widely promoted in some countries.^658^ Many phase III or IV clinical trials were conducted to understand the effect of ivermectin for the treatment of COVID-19. Among them, a double-blind, placebo-controlled, randomized trial involving 476 patients with mild COVID-19 was conducted in Colombia (NCT04405843). According to the data, a 5-day course of ivermectin initiated in the first 7 days after evidence of infection failed to significantly improve the time to resolution of symptoms compared with placebo.^659^ The researchers indicated that this may be due to the relatively healthy and young study population in this trial, highlighting the need to study the ability of ivermectin to prevent more severe COVID-19. Further, a phase III, multicenter, open-label, randomized clinical trial (I-TECH) evaluating the efficacy of ivermectin in 490 high-risk COVID-19 patients was conducted at 20 public hospitals and a COVID-19 quarantine center in Malaysia (NCT04920942). However, researchers concluded that ivermectin treatment during early illness of high-risk patients with mild to moderate COVID-19 did not prevent progression to severe disease.^660^ They also indicated that the open-label trial design might contribute to the underreporting of adverse events in the control group while overestimating the drug effects of ivermectin. Recently, data from a double-blind, randomized, placebo-controlled, adaptive platform trial involving a total of 3515 symptomatic SARS-CoV-2-positive adults recruited from 12 public health clinics in Brazil were published (NCT04727424). In line with previous reports, treatment with ivermectin did not result in a lower incidence of medical admission to a hospital or prolonged emergency department observation for COVID-19 among outpatients at high risk for serious illness.^661^

*Fostamatinib* is approved for treatment of immune thrombocytopenic purpura with potential anti-inflammatory and immunomodulating activities, and its metabolic active form is R406.^662^ Among healthy donor neutrophiles stimulated with COVID-19 patient plasma, treatment with R406 abrogated the release of neutrophil extracellular traps associating with mortality in COVID-19.^663^ Thus, fostamatinib was recognized to be a therapeutic regent for COVID-19, for which a phase II clinical trial was conducted in 60 hospitalized COVID-19 patients (NCT04579393). Results showed that fostamatinib with standard-care treatment decreased the all-cause mortality rate, days on supplemental oxygen, number of days in the ICU, and serious adverse event rate compared with the placebo group.^664^ However, larger randomized clinical trials should be conducted to reliably verify these findings and further investigate the full effects of fostamatinib on inflammation in patients. Thus, multicenter phase III studies evaluating the efficacy and safety of fostamatinib in COVID-19 subjects are underway (NCT04629703; NCT04924660).

*Niclosamide* is an oral anthelmintic drug approved for use against tapeworm infections.^665^ A phase II randomized, placebo-controlled clinical trial showed no significant difference in oropharyngeal clearance of SARS-CoV-2 at day 3 between the placebo and niclosamide groups^666^ (NCT04399356). However, due to the small enrollment pool and unavailable of drug blood levels, further studies should be considered in a wider range of patients. Currently, three phase III clinical studies are in the recruiting status to evaluate its efficacy in COVID-19 patients (NCT04558021; NCT04603924; NCT04870333). A phase IV open label, multi-arm, prospective, adaptive platform, randomized controlled trial involving niclosamide arm and niclosamide in combination with bromhexine arm was completed in June, 2022 (NCT05087381), and the results of the trial are awaiting publication. Since niclosamide is a historically well-tolerated and widely used anthelmintic drug, further escalation studies on this drug will be helpful in the fight against SARS-CoV-2.

*Danoprevir* boosted by ritonavir (Ganovo) is an HCV protease (NS3/4A) inhibitor, which was approved in China in 2018 to treat chronic HCV infection.^667^ According to the data from an open-label, single arm phase IV study in 11 COVID-19 patients (NCT04345276), Chen et al. concluded repurposing it for COVID-19 could be a promising therapeutic option.^668^ According to another study reported by Zhang et al., danoprevir/ritonavir-treated group exhibited shorter time to negative nucleic acid testing and a shorter hospital stay than lopinavir/ritonavir-treated group.^669^ However, given the lack of a placebo control group and the small sample size, further investigation should be conducted to verify this conclusion.

### Candidates under phase I/II clinical trials

Phase I/II clinical trials often focus on drug safety, tolerance, pharmacokinetics, and the benefit/risk ratio in a small number of patients. Currently, many COVID-19 drugs are in these stages (Table 3), and some of them have demonstrated potential in entering a phase III clinical trial.^41^ For example, prostacyclin is a powerful vasodilator and inhibits platelet aggregation. Its sodium salt is used to treat primary pulmonary hypertension. Since endotheliopathy is a prominent feature of COVID-19 and associated with mortality in patients,^670,671^ prostacyclin, which has beneficial effects on the endothelium, might be useful adjunctive therapy for COVID-19 vaculopathy.^672,673^ To determine this effect, a multicenter, randomized phase II clinical trial was conducted in 80 adults with severe COVID-19 requiring mechanical ventilation and severe endotheliopathy (NCT04420741).^674^ No significant difference in the number of days alive without mechanical ventilation within 28 days was observed between the prostacyclin and placebo groups.^675^ Besides, two other phase II clinical trials investigating the potential benefits of prostacyclin in severe COVID-19 patients have been completed, but their results have not yet been published (NCT04445246; NCT04452669). Nezulcitinib (TD-0903) is an inhaled lung-selective inhibitor of JAKs with anti-inflammatory activities. The first study of nezulcitinib in human indicated good tolerance in healthy participants (NCT04402866).^676^ Further, a phase II study evaluating the efficiency, safety, pharmacodynamics, and pharmacokinetics of inhaled nezulcitinib in hospitalized patients with COVID-19-associated acute lung injury and impaired oxygenation was conducted in different countries (NCT04402866). This study was divided into two parts, with 25 and 110 participants, respectively,^222^ and the advanced dosage of 3 mg in Part 1 was applied for further investigation in Part 2. According to the data presented on the ClinicalTrials.gov website, ezulcitinib was associated with lower rates of all-cause mortality and serious adverse events compared to the placebo group.

**Table 3:** Candidates under phase III/IV clinical trials

Agent	Mechanism	Structural category	Stage & region
Desidustat	HIF-PHs inhibitor	Amides	Phase II, Mexico
Voclosporin	Calcineurin inhibitor	Amides	Phase II, Netherlands
Ozanimod hydrochloride	S1PRs modifier	Azoles	Phase II, Canada
Zanubrutinib	BTK inhibitor	Azoles	Phase II, USA
Selinexor	XPO1 inhibitor	Azoles	Phase II, USA, UK, France, Austria, Israel, Spain
Tafenoquine succinate	Mpro inhibitor	Alkaloids	Phase II, USA
Palbociclib	CDK4 inhibitor, CDK6 inhibitor	Amides	Phase II, Germany
Rintatolimod	TLR3 agonist	Nucleoside/ Nucleotide Analogs	Phase II, USA
Crocetin	Enhance the oxygenation of vascular tissue	Terpenoids	Phase II, France
Ibrutinib	BTK inhibitor	Azoles	Phase II, USA, Italy
MIB-626	Nicotinamide adenine dinucleotide regulator modifier	Nucleoside/ Nucleotide Analogs	Phase II, USA
Galidesivir	RdRp inhibitor	Nucleoside/ Nucleotide Analogs	Phase I, Brazil
Maraviroc	CCR5 antagonist	Amides	Phase II, Spain, Mexico
Ambrisentan	ETAR antagonist	Other molecules	Phase II, Spain, UK
Clevudine	RdRp inhibitor	Nucleoside/ Nucleotide Analogs	Phase II, Korea
Melatonin	Melatonin receptor antagonist	Alkaloids	Phase II, USA, Spain
Zenuzolac	p38 MAPK inhibitor	Azoles	Phase II, USA
Iloprost	NF-κB inhibitor	Other molecules	Phase II, Denmark, Qatar
Aprepitant	NK1R antagonist	Azoles	Phase II, USA
Poly ICLC	Immune modulator, TLR3 agonist, Natural killer cells stimulant	Nucleoside/ Nucleotide Analogs	Phase I, Canada
Luminol sodium	TNFα inhibitor, IL6 inhibitor	Amides	Phase II, USA, Bulgaria, France, Hungary, Italy, Romania, Spain
SIR0365	RIP1 inhibitor	Unknown	Phase II, USA, Mexico, Pakistan
Epoprostenol sodium	Platelet aggregation inhibitor, PTGIR agonist	Other molecules	Phase II, USA, Denmark, Qatar, France, Germany, Spain, Switzerland
APX-115	NOX inhibitor	Azoles	Phase II, USA
NLC-V-01	Mpro inhibitor	Unknown	Phase II, Israel
Naltrexone hydrochloride	Opioid receptor μ/κ/δ family antagonist	Other molecules	Phase II, USA
TL-895	NTRK inhibitor	Amides	Phase I, USA
Estradiol	ERs agonist	Steroids	Phase II, Qatar, USA
Liothyronine Sodium	THRA agonist, THRB agonist	Amides	Phase II, Greece
TD-139	Gal-3 inhibitor	Other molecules	Phase II, UK
Methotrexate Sodium	DHFR inhibitor	Amides	Phase II, Brazil
Nafamostat Mesilate	TMPRSS2 inhibitor	Other molecules	Phase II, Korea, Russian
Chlorine dioxide	S protein inhibitor	Other molecules	Phase II, USA, Argentina, Peru
Terevalefim	c-Met/HGFR agonist	Azoles	Phase II, Brazil
Masitinib mesylate	PDGFR inhibitor, FGFR3 antagonist, KIT inhibitor	Amides	Phase II, France, Russia
Uproleselan	SELE inhibitor	Amides	Phase II, USA
Alisporivir	CYPB inhibitor, CYPA inhibitor	Amides	Phase II, France
Hydrogen peroxide	Peroxide and oxidizing agent, Induction of the innate antiviral inflammatory response	Other molecules	Phase II, USA
Asapiprant	PTGDR antagonist	Azoles	Phase II, USA, Argentina, Brazil
IB-MECA	ADORA3 agonist	Nucleoside/ Nucleotide Analogs	Phase II, Bulgaria Romania, Israel
Danicopan	CFD inhibitor	Amides	Phase II, USA
Dactolisib	mTOR-PI3K-AKT pathway inhibitor	Alkaloids	Phase II, USA
Pentarlandir™ UPPTA	Mpro inhibitor	Other molecules	Phase II, USA
Ensifentrine	PDE3 inhibitor, PDE4 inhibitor	Amides	Phase II, USA
Cenicriviroc mesylate	CCR2 antagonist, CCR5 antagonist	Amides	Phase II, Germany
Ebselen	Mpro inhibitor	Azoles	Phase II, USA
Estetrol	Selective estrogen receptor modulator	Steroids	Phase II, Belgium, Hungary, Poland, Russia
Dalcetrapib	CETP inhibitor, HDL cholesterol stimulant	Amides	Phase II, Canada
Arformoterol/ budesonide	ADRB2 agonist, GR agonist	Steroids	Phase II, Brazil
Trans-Sodium Crocetinate	Oxygen compounds modifier	Terpenoids	Phase II, Romania
Quercetin	Free radical scavenger, Mpro inhibitor, PLpro inhibitor	Flavonoids	Phase II, France, USA
Avasopasem	Superoxide dismutase stimulantor	Other molecules	Phase II, USA
Apilimod mesylate	IL12 inhibitor	Nucleoside/ Nucleotide Analogs	Phase II, USA
Emricasan	Apoptosis inhibitor, CASP inhibitor	Amides	Phase I, USA
RP-7214	DHODH inhibitor	Unknown	Phase II, India
VGX-1027	p38 MAPK inhibitor, immunosuppressant, Cytokine inhibitor, NF-κB inhibitor	Azoles	Phase II, USA, Bulgaria, Korea, North Macedonia, Puerto Rico
Nezulcitinib	JAK inhibitor	Other molecules	Phase II, USA, UK, Brazil, Finland, Moldova, Romania, Ukraine
VB-201	TLR2 antagonist	Other molecules	Phase II, Israel
Deupirfenidone	Cytokine inhibitor, Collagen inhibitor	Other molecules	Phase II, USA, Argentina, Brazil, Moldova, Philippines, Romania, Ukraine, UK
Razuprotafib	PTP1B inhibitor, TIE2 antagonist	Amides	Phase II, USA
OP-101	Free radical inhibitor	Amides	Phase II, USA
Zotatifin	EIF4A1 inhibitor	Other molecules	Phase I, USA
Lufotrelvir	Mpro inhibitor	Nucleoside/ Nucleotide Analogs	Phase I, USA, Belgium, Brazil, Spain
Fenretinide	Anti-inflammatory; Antiviral	Terpenoids	Phase II, Canada, USA
Dapansutrile	NLRP3 inhibitor, Interleukin inhibitor, Inflammasome inhibitor	Other molecules	Phase II, USA, Netherlands, Switzerland
Brilacidin	Membrane permeability enhancer	Amides	Phase II, USA, Russia
Bexotegrast	ITGAV&ITGB1 inhibitor, ITGAV&ITGB6 antagonist	Other molecules	Phase II, USA
Enpatoran	TLR7 antagonist, TLR8 antagonist	Other molecules	Phase II, USA, Brazil, Philippines
STC3141	Neutralize NETs/histone	Unknown	Phase II. Belgium
PJS 539	Viral uptake and replication inhibitor	Unknown	Phase II, Brazil
Ezurpimtrostat	Autophagy inhibitor, Apoptotic stimulant	Other molecules	Phase II, France
EC-18	CD4 agonist, CD8 stimulant	Other molecules	Phase II, USA, Korea
UNII-V2YK90BZ31	Immune modulator, HMOX1 inhibitor, Virus replication inhibitor	Other molecules	Phase I, Egypt
Metformin glycinate	PRKAB1 activator, Insulin sensitizer	Other molecules	Phase II, Mexico
ADX-629	Malondialdehyde inhibitor	Alkaloids	Phase II, USA
Bemcentinib	AXL inhibitor	Azoles	Phase II, USA
Sinapultide	Membrane permeability enhancer	Amides	Phase II, USA, Argentina
Brequinar Sodium	DHODH inhibitor	Alkaloids	Phase II, India, USA
Telacebec	Mycobacterium tuberculosis inhibitor, Bacterial growth regulator, Electron transport complex III inhibitor	Amides	Phase II, South Africa
Compound name	Mechanism	Classification of category	Stage & region
CNM-ZnAg	Unknown mechanism of action	Other molecules	Phase II, Brazil
MRG001	CXCR4 antagonist, Calcineurin inhibitor, BMPR2 modifier, Cytokines inhibitor	Unknown	Phase II, USA
Silmitasertib	CK II inhibitor	Other molecules	Phase II, USA
EDP 235	Mpro inhibitor	Unknown	Phase I, USA
INNA 051	Immune stimulant, TLR2 agonist, TLR6 agonist	Amides	Phase II, USA
Idronoxil	SPK 1 inhibitor, SPHK2 inhibitor	Flavonoids	Phase I, Moldova
PHR-160	CFTR activator	Unknown	Phase I, Iran
Ribavirin	RdRp inhibitor	Nucleoside/ Nucleotide Analogs	Phase I, USA, Greece, Mexico
AV-001	Unknown mechanism of action	Amides	Phase I, USA

## Outlook

Small molecules have demonstrated their potential in the development of therapeutics against COVID-19. Viral proteins, host cell components, and immunoregulatory pathways have been identified as effective targets for COVID-19 treatment in regards to the pathogenic mechanisms of SARS-CoV-2. The diverse drug development strategies of small molecules contribute to their effectiveness. Because of global research efforts, some promising compounds, such as remdesivir, baricitinib, and nirmatrelvir/ritonavir, have already been approved or granted EUA in many countries. Moreover, there are more than 20 small molecule candidates in the phase III/IV clinical trial stages, which have the potential to further enrich the family of COVID-19 drugs.

Despite the above achievements, several issues need to be addressed. It is necessary to improve our understanding of SARS-CoV-2 and its lifecycle. The viral components involved in its pathological process must be characterized. The detailed mechanisms of viral replication and interaction with host cells must be elucidated in detail. It is also important to better understand the mechanisms by which the virus dysregulates the host immune system. This knowledge will contribute to the further development of anti-COVID-19 small molecules. SARS-CoV-2 variants are a critical issue. Several variants of concern, such as Alpha (B.1.1.7), Delta (B.1.617.2), and Omicron (B.1.1.529), have led to sustained challenges to the drug development industry. Drug resistance caused by viral mutations prompts us to continue searching for new compounds, targets, and drug combination strategies. Therefore, it is necessary to obtain up-to-date information regarding each variant to understand the structural influences induced by gene mutation. This would speed up and facilitate small molecule development and optimization. Furthermore, researchers should be encouraged to discover more compounds from natural products bearing multiple structural backbones with various activities. Enrichment of these natural backbones will inspire the structural design of potential small molecule drugs. Some findings of small molecules with promising anti-SARS-CoV-2 ability are still limited to the molecular docking simulation stage, while preclinical and clinical experimental evidence is needed to verify their therapeutic properties. Recently, some drugs have shown potential for use in combination therapy in clinical studies.^677,678^ Based on this, researchers should also develop drug combination strategies for existing small molecules to achieve synergistic therapeutic effects. The side effects of each candidate should also be addressed during drug development. In conclusion, the rapid progress in the development of anti-COVID-19 small molecule drugs has definitely strengthened global efforts to combat the SARS-CoV-2 pandemic.
